# Impact evaluation of different cash-based intervention modalities on child and maternal nutritional status in Sindh Province, Pakistan, at 6 mo and at 1 y: A cluster randomised controlled trial

**DOI:** 10.1371/journal.pmed.1002305

**Published:** 2017-05-23

**Authors:** Bridget Fenn, Tim Colbourn, Carmel Dolan, Silke Pietzsch, Murtaza Sangrasi, Jeremy Shoham

**Affiliations:** 1 ENN, Oxford, United Kingdom; 2 Institute for Global Health, University College London, London, United Kingdom; 3 Action Against Hunger, New York, New York, United States of America; 4 Action Against Hunger, Dadu, Pakistan; Harvard University, UNITED STATES

## Abstract

**Background:**

Cash-based interventions (CBIs), offer an interesting opportunity to prevent increases in wasting in humanitarian aid settings. However, questions remain as to the impact of CBIs on nutritional status and, therefore, how to incorporate them into emergency programmes to maximise their success in terms of improved nutritional outcomes. This study evaluated the effects of three different CBI modalities on nutritional outcomes in children under 5 y of age at 6 mo and at 1 y.

**Methods and findings:**

We conducted a four-arm parallel longitudinal cluster randomised controlled trial in 114 villages in Dadu District, Pakistan. The study included poor and very poor households (*n =* 2,496) with one or more children aged 6–48 mo (*n =* 3,584) at baseline. All four arms had equal access to an Action Against Hunger–supported programme. The three intervention arms were as follows: standard cash (SC), a cash transfer of 1,500 Pakistani rupees (PKR) (approximately US$14; 1 PKR = US$0.009543); double cash (DC), a cash transfer of 3,000 PKR; or a fresh food voucher (FFV) of 1,500 PKR; the cash or voucher amount was given every month over six consecutive months. The control group (CG) received no specific cash-related interventions. The median total household income for the study sample was 8,075 PKR (approximately US$77) at baseline. We hypothesized that, compared to the CG in each case, FFVs would be more effective than SC, and that DC would be more effective than SC—both at 6 mo and at 1 y—for reducing the risk of child wasting. Primary outcomes of interest were prevalence of being wasted (weight-for-height *z*-score [WHZ] < −2) and mean WHZ at 6 mo and at 1 y.

The odds of a child being wasted were significantly lower in the DC arm after 6 mo (odds ratio [OR] = 0.52; 95% CI 0.29, 0.92; *p =* 0.02) compared to the CG. Mean WHZ significantly improved in both the FFV and DC arms at 6 mo (FFV: *z-*score = 0.16; 95% CI 0.05, 0.26; *p =* 0.004; DC: *z-*score = 0.11; 95% CI 0.00, 0.21; *p =* 0.05) compared to the CG. Significant differences on the primary outcome were seen only at 6 mo. All three intervention groups showed similar significantly lower odds of being stunted (height-for-age *z-*score [HAZ] < −2) at 6 mo (DC: OR = 0.39; 95% CI 0.24, 0.64; *p <* 0.001; FFV: OR = 0.41; 95% CI 0.25, 0.67; *p <* 0.001; SC: OR = 0.36; 95% CI 0.22, 0.59; *p <* 0.001) and at 1 y (DC: OR = 0.53; 95% CI 0.35, 0.82; *p =* 0.004; FFV: OR = 0.48; 95% CI 0.31, 0.73; *p =* 0.001; SC: OR = 0.54; 95% CI 0.36, 0.81; *p =* 0.003) compared to the CG. Significant improvements in height-for-age outcomes were also seen for severe stunting (HAZ < −3) and mean HAZ. An unintended outcome was observed in the FFV arm: a negative intervention effect on mean haemoglobin (Hb) status (−2.6 g/l; 95% CI −4.5, −0.8; *p =* 0.005). Limitations of this study included the inability to mask participants or data collectors to the different interventions, the potentially restrictive nature of the FFVs, not being able to measure a threshold effect for the two different cash amounts or compare the different quantities of food consumed, and data collection challenges given the difficult environment in which this study was set.

**Conclusions:**

In this setting, the amount of cash given was important. The larger cash transfer had the greatest effect on wasting, but only at 6 mo. Impacts at both 6 mo and at 1 y were seen for height-based growth variables regardless of the intervention modality, indicating a trend toward nutrition resilience. Purchasing restrictions applied to food-based voucher transfers could have unintended effects, and their use needs to be carefully planned to avoid this.

**Trial registration:**

ISRCTN registry ISRCTN10761532

## Abstract 2

A cluster randomised trial reveals cash-based interventions reduce stunting in children from very poor families in Pakistan. 6 months of age is a critical point in alleviating this Bridget Fenn and colleagues reveal.

## Author summary

**Why was this study done?:**

- Cash-based interventions (CBIs) are being increasingly used during humanitarian emergencies as an alternative to food-based interventions to prevent wasting.
- However, due to the lack of evidence available on the impact of CBIs in these settings, it is unclear what are the best ways to implement them.
- There is a lack of evidence to show that CBIs are effective in reducing the risk of wasting in young children in humanitarian aid settings.
- There is limited, and sometimes confusing, evidence about the impact of different CBI modalities on nutritional status, and no evidence of a longer-term impact.
- Our study evaluated the effects of three different CBI modalities on nutritional outcomes in children under five years of age at six months and at one year.

**What did the researchers do and find?:**

- We conducted a four-arm parallel longitudinal cluster randomised controlled trial in 114 villages (2,496 households with 3,584 children) in Dadu District, Pakistan.
- The interventions included two different-sized unconditional grants (standard cash [SC] and double cash [DC]), a fresh food voucher (FFV), and a control group (CG).
- We saw a 48% decrease in the odds of a child being wasted in the DC arm and an improvement in ponderal growth in the FFV (+16 weight-for-height *z*-score [WHZ]) and DC (+11WHZ) arms at six months only.
- We saw a negative impact on haemoglobin status in children in the FFV arm (−2.6 g/l).
- All three CBIs resulted in children having a decreased odds of being stunted and an improvement in linear growth at both six months and one year.

**What do these findings mean?:**

- The amount of cash given in this setting was important in terms of reducing the odds of wasting, but the effects were only seen at the six month time point.
- Regardless of which CBI was received, height growth deteriorated less in the intervention groups than in the CG at six months and at one year, indicating improved nutrition resilience in these children.
- Caution is needed when applying restrictions to food vouchers in order to secure a diverse food basket that provides adequate macro- and micronutrients.

## Introduction

The current global estimate of wasting prevalence is 7.4%, affecting approximately 50 million children under the age of 5 y annually [1]. The World Health Assembly (WHA) 2025 target to reduce and maintain childhood wasting at 5% is unlikely to be met [1]. Globally, attention to child and maternal undernutrition is very high, with agreed targets and impetus through, e.g., the Scaling Up Nutrition (SUN) Movement and the Zero Hunger Initiative, as well as WHA nutrition targets and indicators in the recently framed Sustainable Development Goals. In addition, there is considerable attention being paid to food systems and healthy diets as a potentially sustainable means of preventing high levels of stunting, wasting, and micronutrient malnutrition [2]. According to the 2016 Global Nutrition Report, the overall trend is one of reduction in the prevalence of child undernutrition, though the rate of progress between regions is uneven [3], with the most progress occurring in Asia and the least in sub-Saharan Africa. Asia, however, has the largest numbers of wasted and stunted children [4]. Pakistan presents a particular challenge as the nutritional status of children has shown very little progress over the last 15 y and has, for some nutrition indicators, worsened [5]. This is especially so in Sindh Province, which has the highest prevalence of childhood wasting and stunting in Pakistan [6]. The most recently available population data in Sindh Province indicate that the prevalence of wasting and stunting is 15.4% and 48.0%, respectively, in children under 5 y of age [7]. Levels of anaemia and vitamin A deficiency in Sindh Province have both shown an increase since 2001 [5,6]. In 2011, 73% of children under 5 y of age in Sindh Province were anaemic (haemoglobin [Hb] level < 110 g/l) [6]. Taken together, these statistics indicate an ongoing and serious public health problem.

Previous efforts to improve child and maternal nutrition in Pakistan have been inconsistent, and the coordination needed to develop and implement a coherent nutrition strategy has been weak [5]. However, more recently there have been concerted efforts to develop strategies to tackle undernutrition. For example, the Pakistan Integrated Nutrition Strategy, involving government, bilateral agencies, non-governmental organisations, civil societies, and donors, has been developed, and Pakistan joined the SUN Movement in 2013. Furthermore, the national social safety net system, called the Benazir Income Support Programme (BISP), which uses wealth ranking to select the poorest in the population and provides them with a cash transfer, has started to include child nutrition indicators in its targeting.

Globally, it is believed that cash-based interventions (CBIs), which include cash and food vouchers, offer an alternative to food-based interventions for reducing the risk of wasting during seasonal periods of food insecurity referred to as the “lean season”. Since CBIs are increasingly being incorporated into emergency response programming, more information is needed on the impact of these interventions, particularly where nutrition objectives are established. Whilst there is greater evidence of the impact of CBIs in development aid settings on household dietary diversity and access to health care—through improving household income and protecting household assets [8–10]—there is mixed evidence about whether CBIs improve and protect child growth [9,11], with even less evidence from humanitarian aid settings [12]. As well as this, the available evidence on the effectiveness of different CBI designs in humanitarian aid settings is unclear and potentially conflicting. For example, studies comparing the use of cash versus food vouchers have shown different effects [13,14], while no evidence exists regarding whether different amounts of cash are associated with different levels of impact in preventing wasting in emergencies [15]. Furthermore, no evidence exists on the longer-term impacts of CBIs following an emergency response.

This study compares the nutritional status of children under 5 y of age from households that were allocated to receive either a monthly unconditional cash transfer (one of two amounts), a monthly fresh food voucher (FFV), or a standard package of interventions (the control group [CG]) over six consecutive months. A final round of data was collected 6 mo after the last intervention disbursement to determine any residual impact on nutritional status. We investigated the effect of the different interventions, which were delivered by Action Against Hunger working in Dadu District, Sindh Province, Pakistan, within the context of their Women and Children/Infants Improved Nutrition in Sindh (WINS) programme, funded by the European Union (EU), with further funding from the Directorate-General for European Civil Protection and Humanitarian Aid Operations (DG ECHO). Impact was assessed at two time points: immediately after the final disbursement (at 6 mo) and then 6 mo later (1 y after baseline).

The overarching aim of this study was to evaluate the impact of three CBI modalities on nutritional outcomes in children under 5 y of age from poor and very poor households in Dadu District, Sindh Province, Pakistan, in the context of the lean season. We hypothesized that, compared to the CG, FFVs would be more effective than cash of the same value, and that a higher amount of cash would be more effective than a lower amount of cash at both 6 mo and 1 y in terms of reducing the risk of wasting.

## Methods

### Ethics statement

Ethical approval was obtained from the Pakistan National Bioethics Committee and the Western International Review Board. The trial was registered on 26 March 2015 with the ISRCTN registry (ISRCTN10761532). Participating households were enrolled at baseline after providing written informed consent from the household head or the participating mother, father, or primary carer.

### Study setting

Dadu District is largely agrarian, with the economy dependent on crop production, livestock keeping, and agriculture labour. The majority of the population is highly vulnerable to environmental shocks, especially the poorest households, and there is a lack of alternative income sources, which are further constrained by a lack of economic opportunities. Dadu District experiences frequent flooding and droughts, and extreme temperatures (above 45°C). The results from the most recent nutrition survey, conducted in November 2014 in Dadu District, estimated that 14.3% of children aged 6–59 mo were wasted. For our study, initiated at the start of the lean season (May/June) and including poorer households, we expected the baseline prevalence of wasting to be higher.

### Study design and participants

This was a longitudinal cluster randomised controlled trial, with four parallel arms, conducted among 114 villages, selected from the Action Against Hunger WINS programme database, in Dadu District, Pakistan. The trial design, setting, and characteristics of the study population have been previously described [16].

Households were selected from villages from three agricultural areas sharing similar livelihoods, geography, and access to the same elements of the standard WINS programme. Action Against Hunger provided the initial household lists, and these were further verified and updated by the study research team. Households defined as poor or very poor—using eligibility criteria decided upon by the research team with village participation, and based on ownership of cultivated land and number of goats—and with one or more children aged 6–48 mo were selected. The study was a closed cohort and followed all children in the same eligible households regardless of their baseline anthropometric status.

The study also involved a mixed-methods process evaluation to understand further how intervention implementation may have affected intervention impacts in this setting, and to quantify the causes of any impacts seen. Some of the process evaluation results are presented here, particularly those related to the impacts seen in the study. A further analysis is forthcoming focusing specifically on the pathways that were involved in the main impacts seen.

### Interventions

Three CBIs were implemented: two unconditional cash transfers—a “standard cash” (SC) amount of 1,500 Pakistani rupees (PKR) (approximately US$14) and a “double cash” (DC) amount of 3,000 PKR (approximately US$28)—and one FFV with a cash value of 1,500 PKR (approximately US$14), which could be exchanged for specified fresh foods (fruits, vegetables, milk, and meat) in nominated shops. Action Against Hunger ensured that all FFV villages had good access to these shops, by nominating shops in, or nearby, these villages. All villages were served by at least one nominated shop.

The cash and vouchers were disbursed at the same time every month for six consecutive months. The CG received no additional intervention beyond the basic WINS programme activities (described below) that were provided to all groups. A pure CG was not feasible given WINS programme coverage across Dadu District.

The SC amount was set to equal the amount disbursed by the BISP at the time of the baseline survey. At the time of the study, the purchasing power parity for Pakistan was 0.286 PKR = US$1. The cash and vouchers were disbursed at distribution points on a monthly basis either by mobile banks that travelled to a central location serving some of the participating villages or through central banks that served a number of villages. The FFVs were disbursed to participating households at the village level. All three interventions were delivered with verbal messaging from Action Against Hunger field staff, who were present at all distributions, that children should benefit from the transfers.

All villages had access to the WINS programme, which provided outpatient treatment for children 6–59 mo with severe acute malnutrition (SAM), micronutrient supplementation (children and pregnant and lactating women), and behaviour change communication (BCC). Key BCC messages on the causes of undernutrition, the benefits of exclusive breastfeeding, improved complementary feeding practices, food and water hygiene, handwashing, and sanitation were targeted at mothers. These messages were delivered monthly to all study participants in group sessions by the research mobilisers. Research mobilisers also facilitated data collection activities, such as locating households and setting up times to be available, but were not involved in the data collection itself.

Children identified as severely malnourished during the study period were referred to outpatient treatment. These children were still followed up, with consent from the parents, and were identified in the dataset as to whether or not they received supplementary rations. All parents gave consent, and receipt of supplementary rations was adjusted for in the analysis.

Two of the intervention arms (SC and FFV) were funded by the EU. The DC arm was funded by the DG ECHO. The interventions took place over six consecutive months (July to December 2015).

### Primary outcomes

The primary outcomes were the prevalence of being wasted (weight-for-height *z-*score [WHZ] < −2) and mean WHZ at 6 mo and at 1 y amongst children less than 5 y.

### Secondary outcomes

Secondary outcomes in children were prevalence of SAM (WHZ < −3), mean mid-upper arm circumference (MUAC), prevalence of stunting (height-for-age *z-*score [HAZ] < −2), prevalence of severe stunting (HAZ < −3), mean HAZ, morbidity, mean Hb concentration, and prevalence of anaemia (Hb < 110 g/l) and severe anaemia (Hb<70 g/l) at 6 mo. Due to the longer-term nature of stunting, the stunting outcomes were also assessed at 1 y. Secondary outcomes for mothers were also assessed, including mean Hb concentration and MUAC. Population cutoffs of 120 g/l and 130 g/l for pregnant and non-pregnant women, respectively, were used to determine levels of anaemia. Body mass index (BMI) was assessed for non-pregnant mothers.

### Recruitment

As Global Positioning System mapping is not permitted in Pakistan, the research team carried out a mapping exercise by hand to assess the size of each village and the potential number of eligible households. Only one small village (five households) declined to be included in the study. Because it was not possible to carry out a public randomisation, randomisation was done by the principal investigator (PI) using a random number table to generate the randomisation sequence and then drawing village names from a box. Block randomisation was done, allowing equal distribution of the villages to each arm for small (<40 households), medium (40–85 households), and large (>85 households) villages. The PI had no knowledge of the villages involved and was not involved in the intervention implementation or any data collection. Study participants were enrolled by the data collection team and were not aware which of the interventions they would be getting at enrolment. However, masking of participants was not possible due to the nature of the intervention. The data collection team was different to the cash and voucher disbursement team. The data collection team was responsible for the collection of data and sensitisation of the study recipients to the use of the cash and vouchers. The data collection team was accompanied by local research mobilisers who, as well as facilitating the data collectors in, e.g., locating households, were also responsible for delivering key BCC messages.

### Sample size

The target sample size (approximately 632 households per arm) was calculated to measure a detectable difference of prevalence of being wasted of 7% between the intervention groups and the CG post-intervention [15]. The sample size was also powered to detect a 0.19 WHZ difference between the intervention groups and the CG. This sample size was reached for the SC, FFV, and CG arms. However, for the DC arm the sample size was 600 due to the different funding amounts given for this arm, which did not allow for an equivalent number of households to be included compared to the other three arms. The target sample size was calculated using an estimated intraclass correlation coefficient (ICC) of 0.02 for prevalence of being wasted from an Action Against Hunger nutrition survey in Dadu District. The ICC for prevalence of being wasted for this current study was 0.01.

### Data collection

Quantitative data were collected at baseline and then after each cash and voucher disbursement (6 mo in total), with a final round of data collection 1 y after baseline. Data for Hb were collected (using the HemoCue Hb 201+ System) only at baseline and at 6 mo due to the costs involved. Data for the main impact analysis and findings reported here involved three periods: baseline (May to July 2015), 6 mo after baseline (December 2015), and 1 y after baseline (June/July 2016). Data collected from the months between baseline and 1 y were analysed to illustrate the changes in the prevalence and mean of weight-based indicators during this time. These monthly data will be analysed further in a mediation analysis to be published at a later stage. All questionnaires were translated and administered in the local language, Sindhi. Piloting and back-translation were carried out to ensure that the intended meaning of the questions was retained. Quantitative data were collected using android mobile phones with Open Data Kit software. In order to ensure the quality of the data collected, daily field supervision, meetings with the study coordinator, a mid-term refresher training session, and regular checking of the data were carried out. Data were sent to the ENN PI on a weekly basis for checking.

Qualitative data were collected using focus group discussions (FGDs), key informant interviews, and longitudinal in-depth interviews. Data were collected by a qualified qualitative researcher who conducted two rounds of in-depth interviews with 32 study mothers and 34 FGDs that included study mothers and fathers and other female and male non-participants. Qualitative data were collected using digital dictaphones, and the mp3 files of the recorded interviews were transcribed and translated into English in MS Word and then analysed using a thematic approach. For this analysis, the qualitative data have been used to help interpret the main findings.

### Data processing and statistical analyses.

Data entry and validation checks were conducted both by the research team and ENN. Analysis was conducted entirely by the PI and was supported and verified by a statistical adviser.

The ZSCORE06 command [17] in STATA (SE version 14; StataCorp) was used to calculate *z*-scores. WHZ data were coded as missing if WHZ > +5 or WHZ < −5; HAZ data were coded as missing if HAZ > +5 or HAZ < −6. A child’s data were excluded from the analysis if the child was deemed to be a different child to the child enrolled at baseline. Whilst checks were put in place to ensure that the same child was measured every month, in some cases these were not followed. We used as our criteria for exclusion a decrease in height or length of more than 1 cm (measurement error) or an increase of more than 15 cm (considered the maximum height a child could grow in 6 mo).

Proportions, means (standard deviations), and medians (interquartile ranges) are presented for key baseline variables for households, mothers, and children. All effect analyses are intention-to-treat. Results are presented as crude difference-in-differences estimates (DDEs), adjusted linear changes over the study period, and partially and fully adjusted effect sizes at 6 mo and at 1 y with 95% confidence intervals (CIs). In order to account for clustering at the distribution point level, multilevel mixed-effects regression models were used to generate odds ratios (ORs) for binary outcomes and regression coefficients (β) for continuous outcomes. These results at each time point compare the intervention arm to the CG. The intervention distribution point and household were included as random effects. Village size (small, medium, or large), child age at baseline, child sex, and baseline values of the outcome variables were included as fixed effects in all models. Baseline values of the outcome variables were added into the adjusted models to take into consideration any individual variation at baseline. The village size variable was included to account for different village sizes used for block randomisation; child age and sex and baseline values of the outcome variables were included to adjust for potential individual differences at baseline. Sensitivity analyses (with and without adjustment for other baseline characteristics) were carried out to assess whether adjusting for chance residual baseline imbalances significantly altered the results, such as access to the BISP, deworming, and socio-economic status. These baseline characteristics were those observed as dissimilar in terms of a difference in proportion, mean, or median across arms.

To measure the intervention effect, we included an intervention × time term in the model. Significance was defined as *p <* 0.05. All analyses were carried out in STATA software SE version 14.

## Results

**Fig 1:**
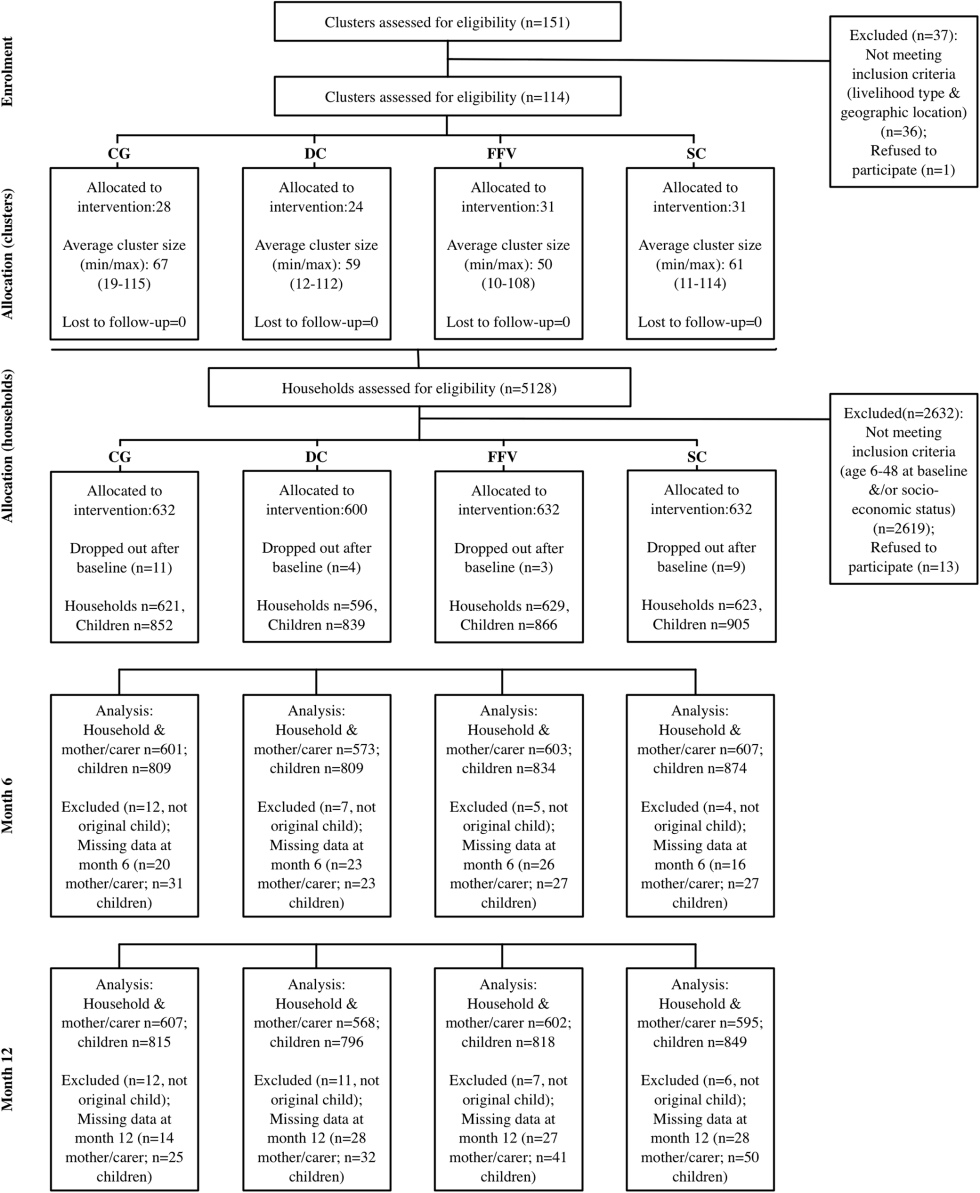
CONSORT flowchart for the study participants. CG, control group; DC, double cash; FFV, fresh food voucher; SC, standard cash.

The flow of clusters and participants through the trial is shown in Fig 1. Enrolment and baseline data collection started together at the end of May 2015 and continued until the beginning of August 2015. Thirteen eligible households refused to participate at the enrolment stage as permission was not given by the head of household. Twenty-seven households migrated away from their village after enrolment (CG = 11, DC = 4, FFV = 3, SC = 9) and were not replaced. These households had similar baseline characteristics between arms. There were a small number of children for whom outcome data were collected who were considered to have been different from the child enrolled at baseline, and these children were excluded from the analysis at 6 mo (*n =* 29) and at 1 y (*n =* 36). Overall, the number of households was slightly lower in the DC arm, which was known before randomisation but was not in the original research protocol. No evaluation clusters were lost to follow-up; response rates for households and children, respectively, within clusters were 95.6% and 98.3% at 6 mo and 95.0% and 96.8% at 1 y. The number of missing child data was slightly lower at 1 y compared to 6 mo for the CG only, as efforts were made to reduce loss to follow-up by offering a hygiene kit once the final data had been collected. Compared to the other arms, the CG had the lowest number of missing child data but, as the extent of missing data was small for all arms, we did not anticipate any effects on comparability between arms. All clusters received and utilised the correct intervention assigned during implementation.

Baseline characteristics of clusters and participants between the different intervention arms and the control arm were well balanced for mothers and their children, apart from the proportion of children who had received deworming treatment, which was lower in the CG (Table 1). There were a few potential imbalances at the household level and between villages due to the clustered nature of the study design. These include village size, ethnicity, access to safe water, and distance to nearest health service. In the CG, there was a higher proportion of households of Balochi ethnicity. In this arm, there also appeared to be differences in the socio-economic status and educational status of mothers and fathers (both lower) and a higher number of households participating in the BISP.

**Table 1:** Baseline characteristics of clusters and individuals by trial arm.

Category	Characteristic	Trial arm			
		Control	Double cash	Fresh food voucher	Standard cash
**Cluster characteristics**	**Number of villages**	28	24	31	31
	**Size of eligible households, median (IQR)**	12 (9–22)	15 (8–28)	16 (10–23)	11 (8–25)
	**Size of village, *n* (percent)**				
	Small	210 (33.8)	129 (21.6)	262 (41.7)	179 (28.7)
	Medium	268 (43.2)	303 (50.8)	231 (36.7)	308 (49.4)
	Large	143 (23.0)	164 (27.5)	136 (21.6)	136 (21.8)
**Household characteristics**	**Number of households**^**a**^	621	596	629	623
	**Ethnicity, *n* (percent)**				
	Sindhi	515 (82.9)	523 (87.8)	612 (97.3)	587 (94.2)
	Balochi	105 (16.9)	59 (9.9)	17 (2.7)	36 (5.8)
	Punjabi	1 (0.2)	14 (2.4)	0	0
	**Muslim religion, *n* (percent)**	621 (100)	592 (99.3)	629 (100)	622 (99.8)
	**Household size, mean (range)**	7.0 (3–22)	7.3 (2–24)	7.4 (2–22)	7.0 (2–20)
	**Wealth category**^**b**^**, *n* (percent)**				
	Most poor	154 (24.8)	129 (21.6)	143 (22.7)	112 (18.0)
	More poor	130 (20.9)	123 (20.6)	145 (23.1)	137 (22.0)
	Poor	106 (17.1)	90 (15.1)	91 (14.5)	114 (18.3)
	Less poor	132 (21.3)	128 (21.5)	113 (18.0)	134 (21.5)
	Least poor	99 (15.9)	126 (21.1)	137 (21.8)	126 (20.2)
	**BISP participation, *n* (percent)**	104 (16.8)	68 (11.5)	59 (9.4)	46 (7.4)
	**Distance to health facility, *n* (percent)**				
	<1 km	14 (2.3)	216 (36.2)	77 (12.2)	129 (20.7)
	1–5 km	318 (51.2)	254 (42.6)	357 (56.8)	219 (35.2)
	>5 km	289 (46.5)	126 (21.1)	195 (31.0)	275 (44.1)
	**Access to safe water**^**c**^**, *n* (percent)**	57 (9.2)	92 (15.4)	49 (7.8)	49 (7.9)
	**Primary education or more, *n* (percent)**				
	Mother	28 (4.5)	66 (11.1)	80 (12.7)	63 (10.1)
	Father	197 (31.7)	198 (33.2)	241 (38.3)	249 (40.0)
	**No self-reported hunger**^**d**^**, *n* (percent)**	470 (75.7)	459 (77.0)	495 (78.7)	516 (82.8)
	**Poor hygiene**^**e**^**, *n* (percent)**	320 (51.5)	291 (48.8)	304 (48.3)	312 (50.1)
**Mother characteristics**	**Age (y), mean (min–max)**	33 (19–64)	33 (18–64)	33 (17–68)	33 (18–78)
	**Height (cm), mean (SD)**	152.5 (5.2)	152.4 (5.7)	152.9 (5.4)	152.4 (5.4)
	**MUAC (cm), mean (SD)**	24.3 (3.2)	24.9 (3.5)	25.2 (3.2)	24.4 (3.4)
	**BMI (kg/m**^**2**^**)**^**f**^**, median (IQR)**	20.0 (18.1–22.7)	20.9 (18.5–24.3)	20.8 (18.5–24.0)	20.4 (18.3–23.5)
	**Dietary diversity**^**g**^**, median (IQR)**	7 (6–8)	7 (6–7)	7 (6–8)	7 (6–8)
	**Moderate/poor self-reported health, *n* (percent)**	481 (77.5)	459 (77.0)	463 (73.6)	454 (72.9)
	**K10**^**h**^	25 (20–29)	21 (17–28)	23 (20–29)	20 (17–26)
	**Haemoglobin (g/l), mean (SD)**	100 (19)	106 (18)	104 (18)	103 (18)
**Child characteristics**	**Number of children**	852	839	866	905
	**Girls, *n* (percent)**	431 (50.6)	429 (51.1)	417 (48.2)	433 (47.9)
	**Age (mo), mean (SD)**	23.4 (11.3)	25.9 (12.0)	26.2 (11.9)	25.6 (12.3)
	**WHZ, mean (SD)**	−1.15 (1.30)	−1.24 (1.28)	−1.08 (1.14)	−1.11 (1.34)
	**Wasted (WHZ < −2), *n* (percent)**	184 (21.9)	198 (24.0)	165 (19.3)	196 (22.0)
	**SAM (WHZ < −3), *n* (percent)**	62 (7.4)	74 (9.0)	46 (5.4)	69 (7.7)
	**HAZ, mean (SD)**	−1.97 (1.75)	−1.79 (1.78)	−2.12 (1.69)	−1.98 (1.65)
	**Stunted (HAZ < −2), *n* (percent)**	437 (51.7)	389 (46.5)	473 (54.9)	457 (50.9)
	**MUAC (cm), mean (SD)**	13.5 (1.2)	13.6 (1.3)	13.8 (1.2)	13.5 (1.3)
	**Dietary diversity**^**i**^**, median (IQR)**	8 (6–9)	7 (6–9)	8 (6–8)	7 (6–8)
	**Diarrhoea**^**j**^^,^^**k**^**, *n* (percent)**	298 (35.0)	229 (27.3)	236 (27.3)	228 (25.2)
	**ARI**^**j**^**, *n* (percent)**	273 (32.2)	332 (39.6)	265 (30.6)	310 (34.3)
	**Fever/malaria**^**j**^**, *n* (percent)**	520 (61.3)	517 (61.7)	488 (56.4)	544 (60.2)
	**Haemoglobin (g/l), mean (SD)**	88 (16)	90 (16)	92 (16)	89 (17)
	**Deworming, *n* (percent)**	38 (4.5)	93 (11.1)	111 (12.8)	125 (13.8)

^a^Excludes 27 households that left the study after baseline enrolment.

^b^Sub-categories of the poor and very poor households included in the study; created using principal components analysis based on literacy (men and women), toilet type, primary building material of house, and various assets owned.

^c^Access to protected/covered water source within 30-min return journey.

^d^Household Food Insecurity Access Scale.

^e^Composite variable including cleanliness of house, animal/human faeces around the house, availability of a hand washing device, and soap use.

^f^Excludes pregnant women.

^g^Score derived from nine food groups.

^h^Kessler Psychological Distress Scale.

^i^Maximum score is 12, adapted from Ruel and Menon [18].

^j^Mother reported during the past 2 wk.

^k^Diarrhoea defined as more than three watery stools per day.

ARI, acute respiratory infection; BISP, Benazir Income Support Programme; BMI, body mass index; HAZ, height-for-age *z*-score; IQR, interquartile range; MUAC, mid-upper arm circumference; SAM, severe acute malnutrition; SD, standard deviation; WHZ, weight-for-height *z*-score.

**Fig 2:**
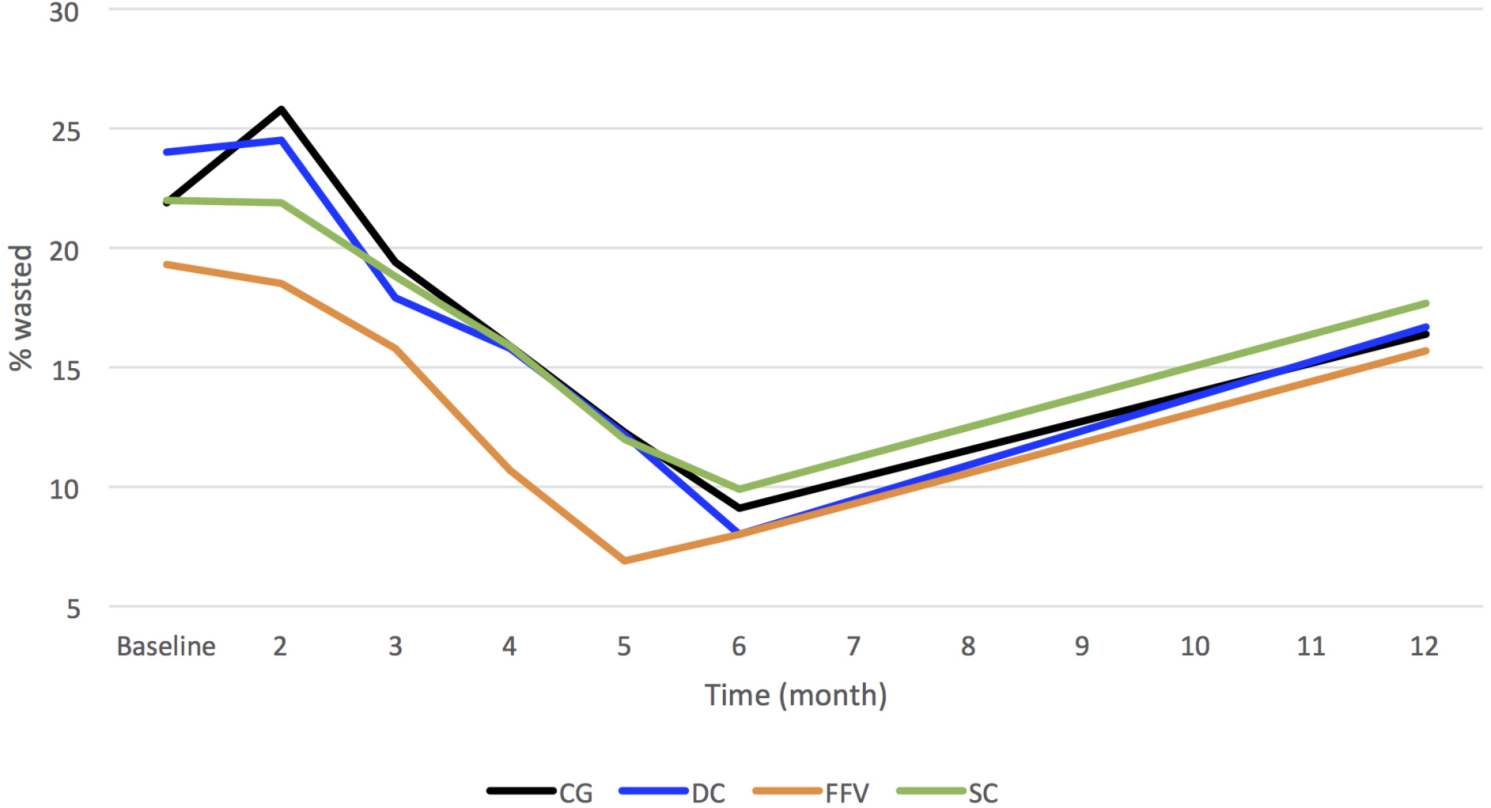
Prevalence of being wasted in children by arm over time. CG, control group; DC, double cash; FFV, fresh food voucher; SC, standard cash.

The proportions of children who were wasted at the different time points are shown in Fig 2. The trend across time was similar for each arm, increasing during the first month and then decreasing to 6 mo. Prevalence was higher again at 1 y, although it was lower than at the same time in the previous year (baseline) for all arms. The differences in prevalence between arms at each month were quite similar.

Assuming a linear change over months (i.e., a consistent rate of change from one month to the next), adjusting for stratification, clustering, and baseline variables, there were no observed significant differences in children being wasted over the 1-y study period (DC: OR = 0.99; 95% CI 0.96, 1.03; *p =* 0.69; FFV: OR = 1.02; 95% CI 0.99, 1.06; *p =* 0.21; SC: OR = 1.02; 95% CI 0.99, 1.05; *p =* 0.29). We were not expecting the interventions to have an immediate effect, but more an accumulative effect, which explains this lack of significance from month to month. From baseline to 6 mo and to 1 y, the largest (unadjusted) DDE in proportion of children wasted was in the DC arm at 6 mo (−3.3%; 95% CI −8.2%, 1.6%, *p =* 0.19), with a smaller reduction at 1 y (−1.6%; 95% CI −7.0%, 3.9%; *p =* 0.58). In both the FFV and SC arms, the DDEs in the proportion of children wasted were higher than in the CG at both time points (FFV—6 mo: 1.6%; 95% CI −3.3%, 6.4%; *p =* 0.53; 1 y: 1.8%; 95% CI −3.6%, 7.2%; *p =* 0.51; SC—6 mo: 0.8%; 95% CI −4.0%, 5.6%; *p =* 0.74; 1 y: 1.0%; 95% CI −4.3%, 6.3%, *p =* 0.71). None of these differences were statistically significant.

**Fig 3:**
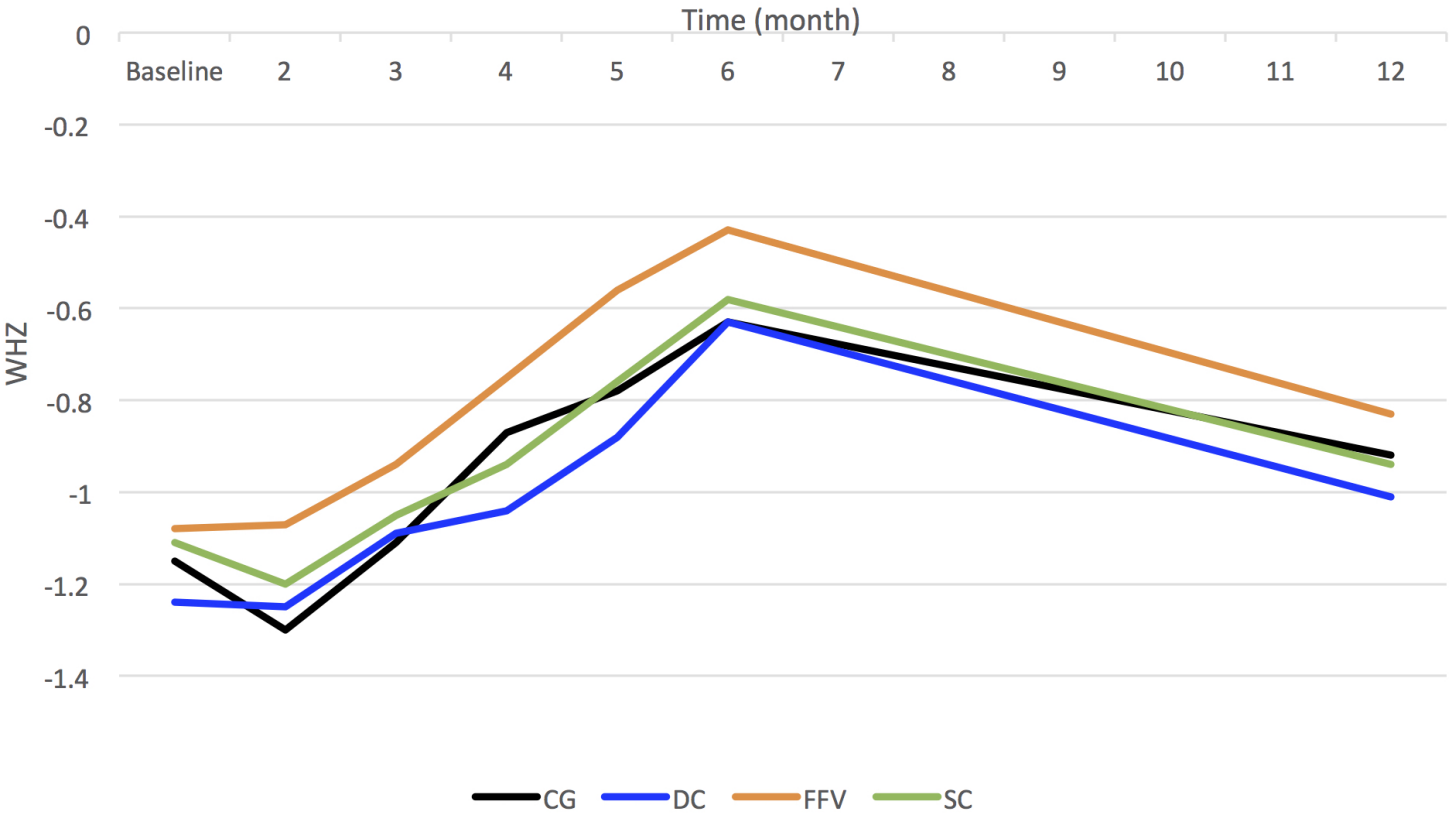
Mean weight-for-height *z-*scores for children by arm over time. CG, control group; DC, double cash; FFV, fresh food voucher; SC, standard cash; WHZ, weight-for-height *z*-score.

Changes in mean WHZ are shown in Fig 3 and show similar trends to the prevalence data in Fig 2. Again, assuming a linear change, adjusting for stratification, clustering, and baseline variables, there are no observed significant differences over months in children’s ponderal growth (DC: −0.003; 95% CI −0.013, 0.007; *p =* 0.62; FFV: −0.002; 95% CI −0.012, 0.008; *p =* 0.74; SC: −0.005; 95% CI −0.015, 0.005; *p =* 0.28).

Between baseline and 6 mo, the largest unadjusted DDE in mean WHZ was in the FFV arm (0.15; 95% CI −0.02, 0.31; *p =* 0.08), followed by the DC arm at 6 mo (0.09; 95% CI −0.07, 0.25; *p =* 0.28). These differences were not statistically significant. The SC arm at both time points and the DC and FFV arms at 1 y showed very little difference from the CG.

The remaining tables show the results from the regression models. The crude models in each case differ from the adjusted models in terms of the width of the CIs, which are narrower in the adjusted models, likely due to inclusion of baseline values of the outcome variables. In nearly all cases, sensitivity analyses resulted in very similar outputs.

The odds of children being wasted in the DC arm were 48% lower compared to the CG at 6 mo (Table 2), adjusted for baseline age, sex, and baseline WHZ. This difference between arms was statistically significant (*p =* 0.02). This intervention effect was seen at 6 mo only and was not observed at 1 y. There were no significant intervention effects for children being wasted in either the FFV or SC arm compared to the CG at either time point.

**Table 2:** Multilevel mixed-effects models estimating odds ratios and regression coefficients (β) for primary outcomes by intervention arm compared to the control group at 6 mo and 1 y.

Outcome variable	Time point and arm	Partially adjusted models^a^		Fully adjusted models^b^	
		OR or β (95% CI)	*p-*Value	OR or β (95% CI)	*p-*Value
**WHZ < −2**	**6 mo**	*n =* 6,680^c^		*n =* 6,532^d^	** **
	DC	0.73 (0.46, 1.16)	0.18	0.52 (0.29, 0.92)	**0.02**
	FFV	1.03 (0.65, 1.63)	0.91	1.16 (0.67, 2.01)	0.6
	SC	1.10 (0.71, 1.70)	0.68	1.09 (0.64, 1.87)	0.75
	**1 y**	*n =* 6,617^e^		*n =* 6,470^f^	** **
	DC	0.90 (0.61, 1.33)	0.6	0.80 (0.51, 1.24)	0.32
	FFV	1.11 (0.75, 1.67)	0.6	1.17 (0.75, 1.82)	0.5
	SC	1.09 (0.74, 1.61)	0.65	1.10 (0.71, 1.71)	0.66
**Mean WHZ**	**6 mo**	*n =* 6,680^c^		*n =* 6,532^d^	
	DC	+0.09 (−0.04, 0.23)	0.19	+0.11 (0.00, 0.21)	**0.05**
	FFV	+0.15 (0.01, 0.28)	0.03	+0.16 (0.05, 0.26)	**0.004**
	SC	+0.03 (−0.11, 0.16)	0.71	+0.04 (−0.07, 0.14)	0.5
	**1 y**	*n =* 6,617^e^		*n =* 6,470^f^	
	DC	−0.01 (−0.15, 0.14)	0.91	+0.00 (−0.12, 0.12)	0.96
	FFV	+0.02 (−0.12, 0.16)	0.77	+0.02 (−0.10, 0.14)	0.79
	SC	−0.08 (−0.22, 0.06)	0.28	−0.08 (−0.19, 0.04)	0.21

Significant *p*-values are shown in bold.

^a^Partially adjusted models adjust for village size and clustering (cluster distribution point and household).

^b^Fully adjusted models also adjust for child age at baseline, child sex, and baseline WHZ.

^c^One hundred nine (1.6%) children missing (WHZ outliers, excluded children, missing data at either time point): CG 31, DC 32, FFV 24 SC 22.

^d^Two hundred fifty-seven (3.8%) children missing (as above): CG 58, DC 83, FFV 54, SC 62.

^e^One hundred twenty-one (1.8%) children missing (as above): CG 33, DC 30, FFV 24, SC 34.

^f^Two hundred sixty-eight (4.0%) children missing (as above): CG 61, DC 84, FFV 56, SC 67.

CG, control group; CI, confidence interval; DC, double cash; FFV, fresh food voucher; OR, odds ratio; SC, standard cash; WHZ, weight-for-height *z*-score.

Children in the FFV arm showed the largest significant increase in mean WHZ in both the partially and fully adjusted models (+0.16 WHZ) at 6 mo, followed by children in the DC arm (+0.11), adjusted for baseline age, sex, and baseline WHZ. These intervention effects were not present at 1 y. The SC arm was no different from the CG for any of the primary outcomes at either time point.

There were no significant changes in the odds of being severely wasted (WHZ < −3) at 6 mo, adjusted for baseline age, sex, and baseline WHZ (Table 3). For mean MUAC, children were no different from the CG for any of the interventions at 6 mo, with ORs very close to zero.

**Table 3:** Multilevel mixed-effects models estimating odds ratios and regression coefficients (β) for key secondary anthropometric outcomes for children by intervention arm compared to the control group at 6 mo and 1 y.

Outcome variable	Time point and arm	Partially adjusted models^a^		Fully adjusted models^b^	
		OR or β (95% CI)	*p-*Value	OR or β (95% CI)	*p-*Value
**WHZ < −3**	**6 mo**	*n =* 6,680^c^		*n =* 6,532^d^	
	DC	0.58 (0.25, 1.34)	0.20	0.37 (0.13, 1.04)	0.06
	FFV	1.04 (0.44, 2.45)	0.92	1.27 (0.45, 3.55)	0.66
	SC	1.12 (0.52, 2.39)	0.77	0.98 (0.38, 2.54)	0.97
**mean MUAC**	**6 mo**	*n =* 6,737^e^		*n =* 6,622^f^	
	DC	−0.01 (−0.14, 0.12)	0.85	−0.06 (−0.15, 0.03)	0.21
	FFV	−0.03 (−0.16, 0.10)	0.69	−0.05 (−0.14, 0.04)	0.27
	SC	0.08 (−0.04, 0.21)	0.20	0.06 (−0.02, 0.15)	0.15
**HAZ < −2**	**6 mo**	*n =* 6,710^g^		*n =* 6,590^h^	
	DC	0.55 (0.38, 0.80)	**0.002**	0.39 (0.24, 0.64)	**<0.001**
	FFV	0.62 (0.42, 0.91)	**0.02**	0.41 (0.25, 0.67)	**<0.001**
	SC	0.62 (0.43, 0.90)	**0.01**	0.36 (0.22, 0.59)	**<0.001**
	**1 y**	*n =* 6,649^i^		*n =* 6,526^j^	
	DC	0.64 (0.45, 0.91)	**0.02**	0.53 (0.35, 0.82)	**0.004**
	FFV	0.62 (0.44, 0.88)	**0.007**	0.48 (0.31, 0.73)	**0.001**
	SC	0.70 (0.49, 0.98)	**0.04**	0.54 (0.36, 0.81)	**0.003**
**HAZ < −3**	**6 mo**	*n =* 6,710^g^		*n =* 6,590^h^	
	DC	0.56 (0.38, 0.84)	**0.005**	0.40 (0.24, 0.68)	**0.001**
	FFV	0.57 (0.39, 0.85)	**0.005**	0.38 (0.23, 0.63)	**<0.001**
	SC	0.66 (0.45, 0.96)	**0.03**	0.47 (0.28, 0.77)	**0.003**
	**1 y**	*n =* 6,649^i^		*n =* 6,526^j^	
	DC	0.74 (0.54, 1.01)	0.06	0.54 (0.34, 0.85)	**0.01**
	FFV	0.75 (0.56, 1.01)	0.06	0.51 (0.33, 0.79)	**0.003**
	SC	0.79 (0.58, 1.07)	0.12	0.59 (0.38, 0.92)	**0.02**
**mean HAZ**	**6 mo**	*n =* 6,710^g^		*n =* 6,590^h^	
	DC	0.25 (0.10, 0.40)	**0.001**	0.24 (0.17, 0.32)	**<0.001**
	FFV	0.26 (0.11, 0.40)	**0.001**	0.27 (0.19, 0.34)	**<0.001**
	SC	0.21 (0.06, 0.36)	**0.005**	0.24 (0.17, 0.32)	**<0.001**
	**1 y**	*n =* 6,649^i^		*n =* 6,526^j^	
	DC	0.22 (0.05, 0.39)	**0.01**	0.21 (0.10, 0.31)	**<0.001**
	FFV	0.29 (0.12, 0.45)	**0.001**	0.29 (0.19, 0.40)	**<0.001**
	SC	0.18 (0.02, 0.35)	**0.03**	0.21 (0.10, 0.31)	**<0.001**

Significant *p*-values are shown in bold.

^a^Partially adjusted models adjust for village size and clustering (cluster distribution point and household). The partially adjusted model for HAZ < −3 at 1 y was not adjusted for household level (model did not converge—fully adjusted model did converge).

^b^Fully adjusted models for WHZ < −3 also include child age at baseline, child sex, deworming, and baseline values of the outcome variables. Fully adjusted models for all other outcome variables also include child age at baseline, child sex, and baseline values of the outcome variables.

^c^One hundred nine (1.6%) children missing (WHZ outliers, excluded children, missing data at either time point): CG 31, DC 32, FFV 24 SC 22.

^d^Two hundred fifty-seven (3.8%) children missing (as above): CG 58, DC 83, FFV 54, SC 62.

^e^Fifty-two (0.8%) children missing (MUAC outliers, excluded children, missing data at either time point): CG 19, DC 16, FFV 9, SC 8.

^f^One hundred sixty-seven (2.5%) children missing (as above): CG 37, DC 58, FFV 32, SC 40.

^g^Seventy-nine (1.2%) children missing (HAZ outliers, excluded children, missing data at either time point): CG 26, DC 19, FFV 19, SC 15.

^h^One hundred ninety-nine (2.9%) children missing (as above): CG 46, DC 61, FFV 44, SC 48.

^i^Eighty-nine (1.3%) children missing (as above): CG 26, DC 21, FFV 19, SC 23.

^j^Two hundred twelve (3.1%) children missing (as above): CG 47, DC 66, FFV 47, SC 52.

CG, control group; CI, confidence interval; DC, double cash; FFV, fresh food voucher; HAZ, height-for-age *z*-score; MUAC, mid-upper arm circumference; OR, odds ratio; SC, standard cash; WHZ, weight-for-height *z*-score.

For other secondary child anthropometric outcomes, all three intervention groups showed a significant decrease in the odds of being stunted and severely stunted and in mean HAZ at both 6 mo and 1 y compared to the CG (Table 3). At 6 mo, the odds of being stunted (HAZ < −2) were 61% (DC) and 64% (SC) lower for the two cash arms, followed by the FFV arm (59% lower odds). For severe stunting, children in the FFV arm had the lowest odds (62% lower), followed by DC (60% lower) and SC (53% lower). At 1 y, the odds of being stunted and severely stunted were similar for the three arms and still statistically significant. Regression coefficients were similar for all three intervention groups at both time points. Children in the FFV arm had the greatest improvement in mean HAZ at both time points (+0.27 and +0.30), followed by similar improvements in the two cash arms: DC (+0.24 and +0.19) and SC (+0.24 and +0.21).

There was no intervention effect on the odds of children being anaemic at 6 mo for any of the intervention arms (Table 4). However, for mean Hb status, children in the FFV arm had a significantly lower Hb level compared to the CG (−2.6 g/l).

**Table 4:** Multilevel mixed-effects models estimating odds ratios and regression coefficients (β) for anaemia and haemoglobin status outcomes for children by intervention arm compared to the control group at 6 mo.

Outcome variable	Arm	Partially adjusted models^a^		Fully adjusted models^b^	
		OR or β (95% CI)	*p-*Value	OR or β (95% CI)	*p-*Value
**Prevalence of anaemia (any)**		*n =* 6,150^c^		*n =* 6,141^d^	
	DC	0.64 (0.39, 1.03)	0.07	0.72 (0.44, 1.19)	0.21
	FFV	1.40 (0.88, 2.21)	0.15	1.42 (0.89, 2.29)	0.14
	SC	0.94 (0.58, 1.53)	0.81	1.13 (0.68, 1.86)	0.64
**Mean haemoglobin**		*n =* 6,150^c^		*n =* 6,141^d^	
	DC	0.17 (−0.03, 0.37)	0.10	0.07 (−0.12, 0.27)	0.48
	FFV	−0.26 (−0.45, −0.07)	**0.008**	−0.26 (−0.45, −0.08)	**0.005**
	SC	−0.01 (−0.20, 0.19)	0.96	−0.12 (−0.31, 0.08)	0.24

Significant *p*-values are shown in bold.

^a^Partially adjusted models adjust for village size and clustering (cluster distribution point and household).

^b^Fully adjusted models also include child age at baseline and child sex. Models not adjusted for baseline haemoglobin status due to the larger number of missing data in the DC and SC arms.

^c^Six hundred thirty-eight (9.4%) children with missing data: CG 48, DC 264, FFV 28, SC 300.

^d^Six hundred forty-seven (9.5%) children with missing data: CG 48, DC 268, FFV 28, SC 303.

CG, control group; CI, confidence interval; DC, double cash; FFV, fresh food voucher; OR, odds ratio; SC, standard cash.

There was no intervention effect for children having diarrhoea for any intervention arm (Table 5). The odds of having an acute respiratory infection (ARI) were 43% lower for children in the DC arm. The odds of having fever/malaria were similarly lower than in the CG for children in both the DC and SC arms (37% and 36%, respectively). The FFVs had no discernable effect on morbidity.

**Table 5:** Multilevel mixed-effects models estimating odds ratios for key morbidity outcomes for children by intervention arm compared to the control group at 6 mo.

Outcome variable	Arm	Partially adjusted models^a^		Fully adjusted models^b^	
		OR (95% CI)	*p-*Value	OR (95% CI)	*p-*Value
**Diarrhoea**		*n =* 6,743^c^		*n =* 6,634^d^	
	DC	0.86 (0.60, 1.24)	0.42	0.87 (0.55, 1.36)	0.54
	FFV	0.97 (0.68, 1.39)	0.87	0.99 (0.64, 1.54)	0.97
	SC	0.98 (0.68, 1.40)	0.90	1.05 (0.67, 1.63)	0.84
**ARI**		*n =* 6,740^e^		*n =* 6,629^f^	
	DC	0.67 (0.49, 0.91)	**0.01**	0.57 (0.40, 0.80)	**0.002**
	FFV	0.89 (0.65, 1.22)	0.49	0.87 (0.61, 1.24)	0.43
	SC	0.78 (0.57, 1.07)	0.12	0.73 (0.51, 1.03)	0.07
**Fever/malaria**		*n =* 6,739^g^		*n =* 6,628^h^	
	DC	0.70 (0.52, 0.94)	**0.02**	0.63 (0.45, 0.89)	**0.01**
	FFV	0.86 (0.64, 1.16)	0.32	0.87 (0.62, 1.22)	0.41
	SC	0.71 (0.53, 0.95)	**0.02**	0.64 (0.46, 0.90)	**0.01**

Significant *p*-values are shown in bold.

^a^Partially adjusted models adjust for village size and clustering (cluster distribution point and household).

^b^Fully adjusted models also include child age at baseline, child sex, and baseline values of the outcome variables.

^c^Forty-six (0.7%) missing data: CG 17, DC 14, FFV 9, SC 6.

^d^One hundred fifty-five (2.3%) missing data: CG 33, DC 54, FFV 32, SC 36.

^e^Forty-nine (0.7%) missing data CG 20, DC 13, FFV 9, SC 7.

^f^One hundred sixty (2.4%) missing data: CG 38, DC 53, FFV 32, SC 37.

^g^Fifty (0.7%) missing data: CG 20, DC 14, FFV 9, SC 7.

^h^One hundred sixty-one (2.4%) missing data: CG 38, DC 54, FFV 32, SC 37.

ARI, acute respiratory infection; CG, control group; CI, confidence interval; DC, double cash; FFV, fresh food voucher; OR, odds ratio; SC, standard cash.

Mothers in the FFV arm saw a significant positive intervention effect on BMI at 6 mo (0.29 kg/m^2^) (Table 6). There was no effect on maternal BMI for the DC or SC arm. There were no effects on mean MUAC across all intervention arms at 6 mo. Mothers were twice as likely to be anaemic in the FFV arm. This negative effect was also seen in the FFV and SC arms for Hb status (−5.0 g/l and −4.2 g/l). There was no intervention effect for anaemia or Hb status for the DC arm at 6 mo.

**Table 6:** Multilevel mixed-effects models estimating odds ratios and regression coefficients (β) for secondary maternal outcomes by intervention arm compared to the control group at 6 mo.

Outcome variable	Arm	Partially adjusted models^a^		Fully adjusted models^b^	
		β or OR (95% CI)	*p-*Value	β or OR (95% CI)	*p-*Value
**Mean BMI**		*n =* 3944^c^		*n =* 3533^d^	
	DC	−0.11 (−0.40, 0.17)	0.43	−0.10 (−0.36, 0.17)	0.47
	FFV	0.29 (0.01, 0.57)	**0.04**	0.29 (0.03, 0.54)	**0.03**
	SC	−0.11 (−0.39, 0.17)	0.44	−0.10 (−0.36, 0.16)	0.45
**Mean MUAC**		*n =* 4786^e^		*n =* 4711^f^	
	DC	−0.17 (−0.40, 0.06)	0.14	−0.18 (−0.40, 0.04)	0.11
	FFV	−0.15 (−0.37, 0.08)	0.20	−0.16 (−0.38, 0.05)	0.14
	SC	0.10 (−0.13, 0.32)	0.41	0.09 (−0.13, 0.30)	0.41
**Prevalence of anaemia (any)**		*n =* 4717^g^		*n =* 4598^h^	
	DC	0.78 (0.54, 1.14)	0.20	0.67 (0.41, 1.07)	0.09
	FFV	1.55 (1.06, 2.25)	**0.02**	2.01 (1.24, 3.27)	**0.005**
	SC	1.17 (0.80, 1.71)	0.42	1.34 (0.82, 2.18)	0.24
**Mean haemoglobin**		*n =* 4717^g^		*n =* 4598^h^	
	DC	−0.05 (−0.27, 0.17)	0.65	−0.09 (−0.30, 0.13)	0.37
	FFV	−0.48 (−0.69, −0.27)	**<0.001**	−0.50 (−0.71, −0.29)	**<0.001**
	SC	−0.38 (−0.60, −0.17)	**<0.001**	−0.42 (−0.63, −0.20)	**<0.001**

Significant p-values are shown in bold.

^a^Partially adjusted models adjust for village size and clustering (cluster distribution point and household).

^b^Fully adjusted models for BMI and MUAC also include socio-economic status and baseline values of the outcome variables; fully adjusted models for anaemia and haemoglobin also include baseline values of the outcome variables.

^c^Nine hundred nine (18.7%) mothers with missing data (women may have been pregnant at either time point, in which case there were no data): CG 222, DC 210, FFV 234, SC 243.

^d^One thousand three hundred seven (26.9%) mothers with missing data: CG 308, DC 315, FFV 334, SC 350.

^e^Sixty-seven (1.4%) mothers with missing data: CG 12, DC 21, FFV 17, SC 17.

^f^One hundred twenty-nine (2.7%) mothers with missing data: CG 24, DC 41, FFV 35, SC 36.

^g^One hundred thirty-six (2.8%) mothers with missing data: CG 24, DC 53, FFV 15, SC 44.

^h^Two hundred forty-two (5.0%) mothers with missing data: CG 30, DC 97, FFV 31, SC 86.

BMI, body mass index; CG, control group; CI, confidence interval; DC, double cash; FFV, fresh food voucher; MUAC, mid-upper arm circumference; OR, odds ratio; SC, standard cash.

### Qualitative study

Whilst households used the different cash amounts in very similar ways: 90% on food, 8% on medical supplies/services, and 2% on non-foods, more households in the SC and FFV arms thought that the 1,500 PKR amount was not enough to meet all their needs:

This money [1,500 PKR] is not enough for us. How can we use it to support the whole family when we have so many children? It finishes quickly because I have a big family. (FGD—SC female)

Everyone has their own needs and one cannot complete these needs from this amount of money [1,500 PKR]. (FGD—SC male)

You know better in this age that nobody can survive with this amount of only 1,500 [PKR]. If you spend 50 rupees every day on vegetables, then that is already 1,500, so it really is a small amount. At the least the voucher should be worth 3,000 [PKR] for our children every month. (FGD—FFV male)

Those getting the 3,000 PKR amount were more content:

We hoped we would get more. However, we are getting 3,000 [rupees], and that is great. (In-depth interview—DC female)

Households receiving either cash amount were happy that they were getting cash rather than the FFVs:

With cash we can buy things as we like or anywhere and at the current market price. With a voucher we can buy things only from one shop, or maybe a few shops, and in redemption our wish is not included. However, with cash we can buy everything including good foods and other items. (FGD—DC male)

If we have cash then we can spend it on any emergency. (FGD—DC male)

And many households receiving the voucher would have preferred the cash:

The token [FFV] is good to buy food only. With cash we can use money in other ways. (FGD—FFV female)

If we don’t have oil to cook with then what sense does it make to have food items. (In-depth interview—FFV female)

We need cash instead of the food voucher. We have our agriculture fields where we can get enough vegetables, such as spinach. (K informant interview—FFV male)

However, some households receiving the FFVs preferred them to cash:

For us the token [FFV] is best because if we have money we will be tempted to spend it on other things. With the token, food will be in our home, so we don’t need to worry. (FGD—FFV female)

The food voucher is best for us because we can only buy vegetables and fruits and nothing else. If you gave us cash, we would use it to buy other things. So the food voucher is the best source, so we can get food items easily and use it to help maintain our childrens’ health. (FGD—FFV male)

However, there was some negative feedback from the FFV arm, especially concerning the vendors where recipient households were able to redeem their vouchers:

The vendors are not good. They don’t give us fresh food. (FGD—FFV female)

They [vendor] give us rubbish [substandard] items; they try to tease us. If we would have money at our hands, they wouldn’t do this. (In-depth interview—FFV female)

They [vendor] charged higher prices in food, for example, against our voucher of 1,500 [PKR]. We received food of only 800 rupees because all the food items have been given to us at higher prices. (FGD—FFV male)

We have one problem from vendors in that they give us foods at high rates as compared to the normal price. (FGD—FFV male)

There was some concern that the vouchers could have been exchanged for cash or other “non-specified” food and non-food items:

No! we never did this. Why would we do this? It is for our children’s health that we didn’t sell it or exchange it with anyone. (FGD—FFV female)

We did think about it, yes. But the vendor said he would give us in exchange 800 rupees—so we didn’t do it. (FGD—FFV male)

## Discussion

Households receiving the larger amount of cash (DC) saw a significant reduction in the odds of their children being wasted at 6 mo. In addition, the DC intervention had positive and significant effects on stunting (HAZ). The FFVs also had positive effects on stunting, although the odds of being wasted for children in this intervention group was no different from that in the CG. No intervention effects for wasting were seen 6 mo after the last disbursement (at 1 y). Children in households receiving SC were no different from children in the CG for the wasting outcome.

All three interventions resulted in a reduction in odds of being stunted and severely stunted and saw positive effects on linear growth (mean HAZ). These effects remained 6 mo after the last disbursement (at 1 y). We did not see any effect from any of the interventions on severe wasting and child MUAC. The FFVs resulted in a reduction in mean Hb concentration, although this did not translate into an increased risk of being anaemic (potentially because the proportion of children already anaemic was very high; approximately 90%). We saw no effects on risk of having diarrhoea for any arm. However, there was a positive intervention effect of DC on ARIs and fever/malaria, and of SC on fever/malaria.

The intervention effects for mothers mirror those for their children for BMI and Hb status: a positive effect on BMI was found for the FFVs and, in the same arm, a negative effect for Hb status, as was the case with the SC arm. For FFVs, this lower Hb status also translated into a significant increase in the risk of being anaemic. As for children, no intervention effects were found for mother’s MUAC status.

The results for the DC arm support our hypothesis that larger amounts of cash combined with BCC can benefit child growth. The qualitative study suggests that households were happier with the larger amount, and this may in itself have conferred a more positive attitude toward the intervention, and potentially toward the uptake of BCC messages. It is interesting, however, that increasing the amount did not confer positive intervention effects on Hb status or anaemia prevalence. There is evidence that CBIs can improve anaemia status [19], although it is possible that in contexts where very high levels of anaemia already exist, non-food/dietary-based factors, e.g., intestinal worm infestation, may mask or undermine any positive impact of the CBIs, suggesting the need for additional interventions in tandem with an increased cash amount.

A surprising and unintended outcome was the significantly lower Hb levels in children and their mothers in the FFV arm. In addition, mothers in the FFV arm saw a significant increase in the prevalence of anaemia. We had thought that households had been allowed to exchange their FFVs—against protocol—for other items such as foods with low levels of iron (rice, oil, or sugar) or foods with detrimental effects on iron absorption (milk, eggs, or tea). The qualitative data, however, confirmed that the vendors rarely exchanged the vouchers for other, non-fresh-food items. Nor did vendors exchange the vouchers for cash. We had hypothesized that, compared to the CG, the FFVs, with a similar value to the SC transfers, would deliver a greater nutrition impact. This was true in terms of WHZ, whereby children grew more in the FFV arm than in the SC arm. However, it was thought that the FFVs would impact growth and micronutrient status through increasing dietary diversity. An analysis of dietary diversity at the mother and child levels (S1 Table) saw a significant improvement for all three arms, but this improvement was lowest in the FFV arm (highest in the DC arm). Regarding child dietary intake of specific foods, it is not obvious if this had an effect on Hb in the FFV arm as, in all significant cases, children in all the intervention arms had a higher intake of specific foods compared to the CG (S2 Table). Whilst there was a significant increase in consumption of animal protein compared to the CG, the type of meat was not differentiated. Qualitative evidence suggests that the only meat available for the FFVs was chicken, which is itself low in iron. The DC arm had higher intakes of both iron-rich foods and iron absorption inhibitors (e.g., milk and eggs), which may explain why increases in Hb were not seen here. Given that child mean WHZ improved over time in the FFV arm, and yet child dietary diversity was lower compared to the CG, a possible explanation is that there were differences in the amounts of food consumed (i.e., children’s caloric intakes could have been higher in the FFV arm). However, it is not possible to conclude anything about the quantities of food eaten from this study, as these data were not collected.

The intervention effect for the DC arm on child weight-based variables was only apparent at 6 mo. This suggests that where CBIs have an objective to reduce the risk of wasting, this can be effective, but when the causes of food insecurity and high morbidity are not removed, children remain vulnerable to wasting. The limited evidence of impact of CBIs on wasting in the literature is entirely focused on the short term [14], unlike for food-based interventions, where there is some evidence that the risk of being wasted remains 12 mo after recovery [20].

For height-based variables, the positive intervention effect was found at both 6 mo and 1 y in all three intervention groups compared to the CG. This is in itself an important finding, as stunting is a well-accepted marker of overall national development, and its reduction to 20% is a WHA target. Many governments, development partners, and global actors are actively supporting efforts to see acceleration in the rates of stunting reduction. High rates of child stunting also carry a mortality risk, and the more severe the stunting, the greater the risk. Moderately stunted children have a 2.3 times increased risk of death, and severely stunted children are 5.5 times more likely to die [21]. In this study, we saw similar and significant improvements in reduced odds of moderate and severe stunting across the intervention arms at 6 mo and 1 y. The finding that the odds of being stunted were significantly reduced in children at 6 mo is a potentially unexpected outcome from this study, given the short-term nature of the interventions. What is reassuring are the similar results at 1 y, indicating a real effect. Another cash-based longitudinal cluster randomised controlled trial set in Malawi also found a positive impact on linear growth over 1 y and attributed this reduction in prevalence of stunting to the intervention improving food security and dietary diversity [22], which we also saw in our study. The results from our study will be further examined in a forthcoming mediation analysis.

The higher amount of cash (DC) reduced the risk of ARIs, and both the SC and DC interventions reduced the risk of fever/malaria. The morbidity reduction effect was stronger in the DC arm and may in part be explained by the improvement in nutritional status of children in this arm. Expenditures on health and access to health services will be evaluated in further analyses. That there was no reduction of risk for any disease in the FFV arms suggests that FFVs are not as effective as cash at reducing morbidity risk, particularly for fever/malaria.

This is one of only two robust RCTs to our knowledge carried out in a humanitarian aid setting showing significant CBI intervention effects on child nutritional status. A similar positive intervention effect using a CBI has been seen in one other published study [23]. Our study provides evidence to inform policy and programmes and offers good practice-based evidence for all those concerned with reducing the risk of increased child undernutrition in emergencies and in severe lean season contexts through CBIs. That the BISP and other programmes in Pakistan have shown great interest in the study and its results provides impetus for future CBI programme design linked with nutrition policy objectives within Pakistan.

The strengths of this study include its randomised design, adherence to the implementation process, good retention rates, extensive process evaluation, and cost-effectiveness analysis (to be published elsewhere). Even though data were collected under difficult conditions, the rate of missing data was low. The theory of change for the relationship between CBIs and nutritional status is complex, making scale-up difficult unless some of the questions about how and why the intervention worked, or didn’t work, are understood. The process evaluation undertaken during this study will be used together with a mediation analysis to understand the “how” and “why” of the intervention effects in a future analysis. The recent High Level Panel on Humanitarian Cash Transfers agreed that cash can be effective in humanitarian aid settings but may, at some times and in some places, be inappropriate [24]. To ensure that CBIs are designed in the best possible way, it is important to ensure that there are functioning markets and to understand the causes of undernutrition within a setting. In Pakistan, a previous nutrition causal analysis identified that low income was a significant underlying cause of undernutrition [25], and the current study has shown that cash transfers can have a positive impact on this underlying cause.

There are a number of limitations to this study. First, masking of the interventions to both participants and data collectors was not possible in this setting and for this type of study. Precautions were taken at the start of the study to try to mask the different interventions to participants, e.g., through incorporating “buffer” zones and training data collectors to keep the information to themselves, but it soon became clear that participants were aware of the other interventions. This was especially so for the CG as the dropout rate for this arm increased more in subsequent months after baseline. However, to encourage continued participation, this group was given a hygiene kit after the last round of data collection. For all groups, the data collectors were trained to sensitise the participants to the study objectives and to ensure the same key messages were highlighted during data collection. The data collectors could not be masked about which arms were getting what intervention because part of the process evaluation was to ask questions about the use of the intervention. To ensure similarity between intervention arms, data collectors were rotated so they covered different groups. The disbursement of the cash and vouchers was done by different organisations, and the cash participants had further to travel to their distribution point, which may well have added to the opportunity costs to households and reduced the actual transfer value. Added to this, the FFV arm had more direct contact with Action Against Hunger field staff during voucher disbursement, which could have affected the results through greater exposure to key messages. Efforts were made throughout the study to engage with the Action Against Hunger field staff and to sensitise them to the study objectives. It is also possible that the vouchers themselves were too restricted. They were designed to purchase fresh fruit, vegetables, and fresh meat and were, therefore, dependent on what the vendors stocked, such as chicken being the only available meat. There were also many anecdotal reports regarding vendors overcharging for food items redeemed against the vouchers as a way to cover their own administration fees in recovering the voucher costs. In this respect, the actual transfer value given may have been lower than the face value.

With these data, it is not possible to calculate a “threshold” for the minimum amount of cash that would have had a significant effect. We know, however, that this threshold falls somewhere between the amounts in the SC and the DC interventions. We can also not say anything about the quantities of food bought or the quality of medical services accessed as these data were not collected. Finally, the Sindh Province context presented a number of difficulties affecting data collection. The baseline survey took longer than expected since recruitment of female data collectors was difficult and was a reason why the baseline data collection was extended. Added to this, temperatures reached 52°C, which not only affected the data collection team’s working ability but also had an effect on the HemoCue devices used to measure Hb. There are more missing data, therefore, for Hb in the two cash arms than in the FFV and CG arms for both children (Table 4) and their mothers (Table 6).

The results from this study are the first to our knowledge to be seen from a CBI programme in a humanitarian aid setting in Asia. Whilst these results are very compelling, the findings raise questions about the optimal approach when using FFVs in contexts of high anaemia prevalence and the need for future programme design to ensure such interventions enable access to the correct foods, in the correct amounts, and do not have restrictions attached to them. At the same time, understanding and mitigating the non-food causes of anaemia are warranted.

### Conclusion

Unconditional cash transfers of at least 3,000 PKR, equivalent to approximately US$28 (twice as much as the SC amount based on the BISP), were more effective in improving weight-based growth immediately following the intervention in a population of poor and very poor households with young children. This effect was seen against a backdrop of very high wasting at baseline (>20%), an indicator of the deleterious effects of seasonal food shortages and high morbidity in this region. The type of intervention did not really matter for height-based variables as all three intervention groups had a significant improvement at both the 6 mo and 1 y time points compared to the CG, thus indicating movement toward greater nutrition resilience, whereby having a better nutritional status increases the capacity of a person or population to withstand shocks or stressors that might adversely affect the causes of undernutrition.

The FFVs had an unintended negative impact on Hb status, and this may have been due to the restrictive nature of the voucher—in this sense, unconditional cash transfers were better than vouchers, though mean WHZ did improve in the FFV arm.

## Supporting information

S1 CONSORT Checklist (DOC)

S1 Table Change in mother and child dietary diversity scores between baseline and 6 mo. Adjusted for village size and clustering (cluster distribution point and household).
(DOCX)

S2 Table Change in child dietary intake between baseline and 6 mo. Adjusted for village size and clustering (cluster distribution point and household); *n* = 6,778^a^.
(DOCX)

S1 Text Protocol. (PDF)

S2 Text Ethical statement. (PDF)

S3 Text Questionnaires. (PDF)

## Abbreviations

ARI: acute respiratory infection
BCC: behaviour change communication
BISP: Benazir Income Support Programme
BMI: body mass index
CBI: cash-based interventions
CG: control group
CI: confidence interval
DC: double cash
DDE: difference-in-differences estimate
DG ECHO: Directorate-General for European Civil Protection and Humanitarian Aid Operations
EU: European Union
FFV: fresh food voucher
FGD: focus group discussion
HAZ: height-for-age *z*-score
Hb: haemoglobin
MUAC: mid-upper arm circumference
OR: odds ratio
PI: principal investigator
PKR: Pakistani rupees
SAM: severe acute malnutrition
SC: standard cash
SUN: Scaling Up Nutrition
WHA: World Health Assembly
WHZ: weight-for-height *z*-score
WINS: Women and Children/Infants Improved Nutrition in Sindh
