# Supplementary material for: Impact evaluation of different cash-based intervention modalities on child and maternal nutritional status in Sindh Province, Pakistan, at 6 mo and at 1 y: A cluster randomised controlled trial
Source: PLoS Med. 2017 May 23;14(5):e1002305. doi: 10.1371/journal.pmed.1002305 (PMC5441577; doi:10.1371/journal.pmed.1002305)
Supplement: S1 Table — Adjusted for village size and clustering (cluster distribution point and household). (DOCX) [file pmed.1002305.s002.docx]

**S1 Table**

Table 1: Mother and child dietary diversity scores between baseline and month 6 (adjusted for village size and clustering (cluster distribution point and household)

| **Dietary diversity** | | |
| --- | --- | --- |
|  | **β (95% CI)** | **p value** |
| **Mother^a^**  **n=4853** |  |  |
| DC | +1.70 (1.48, 1.92) | ***<0.001*** |
| FFV | +0.76 (0.54, 0.98) | ***<0.001*** |
| SC | +1.01 (0.79, 1.23) | ***<0.001*** |
| **Child^b^**  **n=6778^c^** |  |  |
| DC | +0.73 (0.53, 0.93) | ***<0.001*** |
| FFV | +0.43 (0.23, 0.63) | ***0.001*** |
| SC | +0.59 (0.39, 0.79) | ***<0.001*** |

**^a^** Score derived from 9 food groups

^b^ Score adapted from Ruel and Menon [1]

^c^ 10 children with missing data at 1 time point

[1] Ruel MT & Menon P (2002) Creating A Child Feeding Index Using the Demographic and Health Surveys: An Example from Latin America. FCND Discussion Paper no. 130
