# Supplementary material for: Impact evaluation of different cash-based intervention modalities on child and maternal nutritional status in Sindh Province, Pakistan, at 6 mo and at 1 y: A cluster randomised controlled trial
Source: PLoS Med. 2017 May 23;14(5):e1002305. doi: 10.1371/journal.pmed.1002305 (PMC5441577; doi:10.1371/journal.pmed.1002305)
Supplement: S2 Table — Adjusted for village size and clustering (cluster distribution point and household); n = 6,778a. (DOCX) [file pmed.1002305.s003.docx]

**S2 Table**

Table 2: Child dietary intake between baseline and month 6 (adjusted for village size and clustering (cluster distribution point and household); n=6778^a^

| **Dietary intake** | | |
| --- | --- | --- |
|  | **DDE % (95% CI)** | **p value** |
| **Animal protein** |  |  |
| DC | +26.5 (20.6, 32.5) | ***<0.001*** |
| FFV | +20.0 (14.1, 25.8) | ***<0.001*** |
| SC | +18.8 (13.0, 24.6) | ***<0.001*** |
| **Iron rich (organ) meat** |  |  |
| DC | +8.1 (4.7, 11.7) | ***<0.001*** |
| FFV | +1.8 (-1.6, 5.3) | 0.30 |
| SC | +6.0 (2.5, 9.4) | ***0.001*** |
| **Eggs** |  |  |
| DC | +21.0 (15.6, 26.3) | ***<0.001*** |
| FFV | +13.8 (8.6, 19.1) | ***<0.001*** |
| SC | +13.9 (8.7, 19.1) | ***<0.001*** |
| **Milk** |  |  |
| DC | +7.8 (3.6, 12.0) | ***0.004*** |
| FFV | +5.0 (0.9, 9.2) | ***0.02*** |
| SC | +2.8 (-1.3, 6.9) | 0.18 |
| **Vitamin A rich foods** |  |  |
| DC | +6.2 (2.3, 10.2) | ***0.002*** |
| FFV | -1.6 (-5.5, 2.4) | 0.43 |
| SC | +8.5 (4.6, 12.4) | ***<0.001*** |

^a^ 10 children with missing data at one time point
