# Supplementary material for: Impact evaluation of different cash-based intervention modalities on child and maternal nutritional status in Sindh Province, Pakistan, at 6 mo and at 1 y: A cluster randomised controlled trial
Source: PLoS Med. 2017 May 23;14(5):e1002305. doi: 10.1371/journal.pmed.1002305 (PMC5441577; doi:10.1371/journal.pmed.1002305)
Supplement: S1 Text — (PDF) [file pmed.1002305.s004.pdf]

Fenn et al. BMC Public Health (2015) 15:1044
DOI 10.1186/s12889-015-2380-3

BMC Public Health

STUDY PROTOCOL Open Access

The REFANI Pakistan study—a cluster randomised controlled trial of the effectiveness and cost-effectiveness of cash-based transfer programmes on child nutrition status: study protocol

Bridget Fenn[1*], Ghulam Murtaza Sangrasi[2], Chloe Puett[3], Lani Trenouth[3] and Silke Pietzsch[3]

Abstract

Background: Cash-based transfer programmes are an emerging strategy in the prevention of wasting in children, especially targeted at vulnerable households during periods of food insecurity or during emergencies. However, the evidence surrounding the use of either cash or voucher transfer programmes in the humanitarian context and on nutritional outcomes is elusive. More evidence is needed not only to inform the global community of practice on best practices in humanitarian settings, but also to help strengthen national mitigation responses.

Methods/Design: The Research for Food Assistance on Nutrition Impact Pakistan study (REFANI-P) sets out to evaluate the impact of three cash-based interventions on nutritional outcomes in children aged less than five years from poor and very poor households in Dadu District. This four-arm parallel cluster randomised controlled trial is set among Action Against Hunger (ACF) programme villages in Dadu District, Sindh Province. Mothers are the target recipients of either seasonal unconditional cash transfers or fresh food vouchers. A comparison group receives 'standard care' provided by the ACF programme to which all groups have the same access. The primary outcomes are prevalence of wasting and mean weight-for-height Z-score (WHZ) in children. Impact will be assessed at 6 months and at 1 year from baseline. Using a theory-based approach we will determine 'how' the different interventions work by looking at the processes involved and the impact pathways following the theory of change developed for this context. Quantitative and qualitative data are collected on morbidity, health seeking, hygiene and nutrition behaviours, dietary diversity, haemoglobin concentration, women's empowerment, household food security and expenditures and social capital. The direct and indirect costs of each intervention borne by the implementing organisation and their partners as well as by beneficiaries and their communities are also assessed.

Discussion: The results of this trial will provide robust evidence to help increase knowledge about the predictability of how different modalities of cash-based transfer work best to reduce the risk of child wasting during a season where food insecurity is at its highest. Evidence on costing and cost-effectiveness will further aid decisions on choice of modality in terms of effectiveness and sustainability.

Trial registration: Current Controlled Trials ISRCTN10761532. Registered 26 March 2015.

Keywords: Cash transfer, Fresh food voucher, Wasting, Children, Cost-effectiveness, Process evaluation, Sindh province, Pakistan, Research protocol

* Correspondence: bridget@ennonline.net
[1]Independent consultant for the Emergency Nutrition Network, Oxford, UK
Full list of author information is available at the end of the article

BioMed Central

© 2015 Fenn et al. **Open Access** This article is distributed under the terms of the Creative Commons Attribution 4.0 International License (http://creativecommons.org/licenses/by/4.0/), which permits unrestricted use, distribution, and reproduction in any medium, provided you give appropriate credit to the original author(s) and the source, provide a link to the Creative Commons license, and indicate if changes were made. The Creative Commons Public Domain Dedication waiver (http://creativecommons.org/publicdomain/zero/1.0/) applies to the data made available in this article, unless otherwise stated.

Background

Cash-based transfers are fast becoming a standard part of the humanitarian agency toolbox. There is growing interest and use in providing cash or vouchers to vulnerable households who have children at risk of undernutrition during periods of food insecurity or during emergencies [1–4] During these periods the prevalence of acute malnutrition or wasting, due directly to transient nutritional deprivation from deficits in energy intake and/or poor appetite, malabsorption, or loss of nutrients due to disease, will increase unless there are sufficient, and appropriate, mitigation strategies to protect these children. Increased focus on prevention strategies to reduce the global burden of wasting has motivated the use of alternative mitigation approaches such as the use of cash-based transfers [5]. However, there is insufficient empirical evidence to demonstrate that cash-based transfers are an appropriate substitute for food-based interventions to prevent acute malnutrition in children and to understand the circumstances under which these interventions are likely to be effective.

Cash-based transfers can be made as direct cash payments or bank transfers using various modalities such as smart cards, mobile phones, e-wallets and e-vouchers or paper vouchers. Vouchers can be exchangeable for fixed quantities of specific food or non-food items or a service (commodity vouchers), or for cash value - exchangeable for a choice of specified food or non-food items with the equivalent value of the voucher (cash or value vouchers) [6, 7]. It is hypothesised that cash or vouchers can tackle wasting through a number of different pathways [Fig. 1]. However, determining which of these pathways is more important is highly context-specific and evidence surrounding the impact of cash and vouchers on child wasting in humanitarian situations is elusive. As a complex public health intervention the causal density between a cash-based transfer programme and nutrition status is high. There are numerous intermediary factors that make it difficult to know with any predictability how a programme will work. As well as this, very little is known about how cash-based transfers interact with the context in which they are set. Understanding the underlying mechanisms in the causal pathway between cash-based transfers and nutrition status, and a context, is crucial to understanding why an intervention works or not and is necessary in providing useful insights into how the outcomes from such studies could be transferred across different settings and populations.

Most of the available evidence on seasonal cash and food vouchers compares cash and/or vouchers to food transfers with some evidence on cost-efficiency. Also the main focus has been on food access and utilisation [4], rather than nutritional outcomes. This is thought to be due largely to the lack of nutrition objectives [8, 9] because cash-based transfer programmes are typically designed and implemented with food assistance objectives only [10].

A recent four-country study showed that either cash or a food voucher had a greater impact on improving dietary diversity, and were more cost-effective, compared to in kind food transfers [11]. Further evidence has shown that food vouchers can be more effective than cash grants in improving dietary diversity, as well as being more cost-effective [12]. However, another study in the Democratic Republic of Congo showed no observable differences between households receiving either cash grants or vouchers in food security, household coping strategies or asset ownership, though households receiving cash grants reported greater savings [13]. In terms of cost-efficiency, costs associated with delivering cash and voucher transfers have shown to be considerably lower than food in kind transfers [12, 14, 15], with the implementation of cash grants shown to be more cost-efficient than voucher programmes [13].

Cost-effectiveness is an important measurement of program resource use, complementing evidence on intervention effectiveness and providing valuable economic insights to guide decisions regarding resource allocation and priority setting [16, 17]. At present, there is no robust evidence that compares either cash or voucher programmes on nutritional outcomes or longer-term cost-effectiveness regarding the number of cases of malnutrition averted. The REFANI-P study uses a cluster randomised study design to compare three cash-based transfer modalities in Pakistan and to address the evidence gaps on the effectiveness and cost-effectiveness of cash-based transfers on nutritional outcomes.

Methods/Design

Aims and objectives

The overarching aim of REFANI-P is to evaluate three cash-based transfer modalities on nutritional outcomes in children aged less than five years from poor and very poor households in Dadu District, Sindh Province, Pakistan.

This study aims to (1) compare the nutrition status of children receiving either a seasonal unconditional cash transfers or a fresh food voucher with those with access to ACF care only, after 6 months and at 1 year; (2) assess the costs and cost-effectiveness of the different interventions; (3) understand the factors that determine the ways in which households use the different transfers; and (4) explore the role of the different processes involved in the study outcomes and how they interact with the context.

Study design

This is a longitudinal cluster randomised controlled trial (cRCT), with 4 parallel groups with integral process and

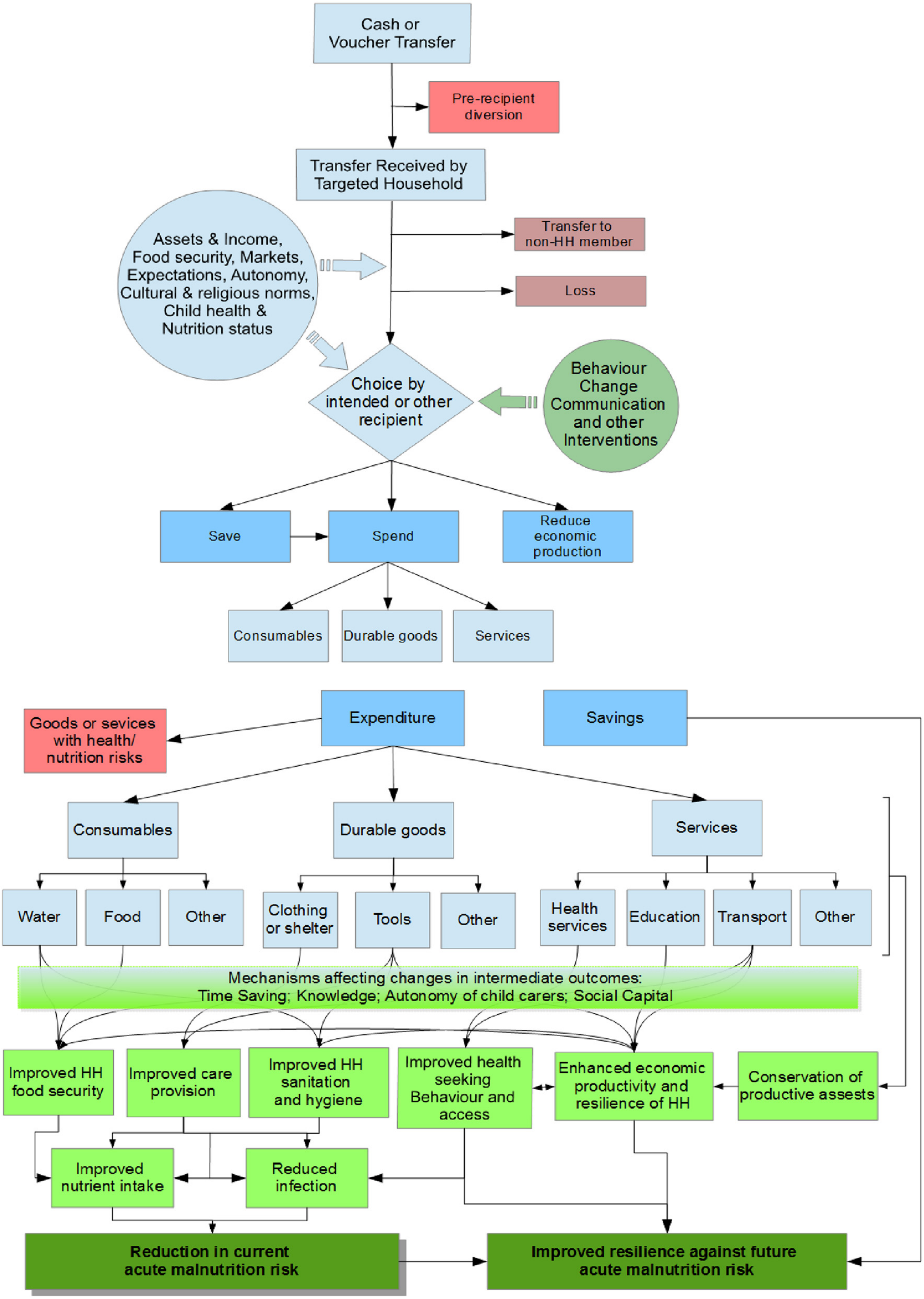

Fig. 1 REFANI Theory of Change

economic evaluations. Cluster assignment is by village. This study follows the same group (closed cohort) of eligible households with children (6–48 months at baseline) in each group over one year. The design is one-stage and all eligible children within eligible households are included. The intervention takes place over 6 consecutive months (June to November 2015). Data are collected at baseline (pre-intervention), then each month during the 6 month intervention period, followed by a final data collection at 1 year (6 months post intervention). Eligible children are the unit for analysis and clustering within villages is taken into account.

The study is also designed to understand the factors that determine the ways in which households use the different transfers and explore the role of the different processes influencing the intervention outcomes and how they interact with the context. This is done using a mixed-methods approach within a process evaluation framework developed for cRCTs proposed by Grant et al. [18]. Qualitative data are also being collected to inform data collection tool design (e.g. by informing content and rational piloting) and to help to understand any associations found (or not) within the quantitative analysis.

The study will also provide evidence on the costs of these cash-based interventions, and their cost-effectiveness in preventing cases of acute undernutrition. The study uses qualitative and quantitative methods to assess intervention resource use from a societal perspective, including all direct and indirect costs of the intervention regardless of who incurs them, including implementing organisation, beneficiaries, or partners etc. Cost-effectiveness ratios are calculated using cost data along with outcome data from each intervention arm.

Ethics and consent

Ethical approval was obtained from the Pakistan National Bioethics Committee on 12th February 2015 (reference number 4-87/14/NBC-170/RDC/2304). The study has approval by the Western International Review Board (11/03/2015). The trial was registered on 26th March; ISRCTN10761532.

Participating households are enrolled after providing written informed consent. The consent form, translated into Sindhi, sets out to explain the study and the procedures. There are no risks associated with participating in REFANI-P and households are informed that they could drop out at any time without any penalty or loss of any benefits from the programme. Confidentiality is guaranteed during the study and no individual quantitative data is released. For the qualitative study permission is gained leading to any publication of individual data collected (including photographs).

Study setting

The study is set in Dadu District in Sindh Province. The economy in Dadu district is largely agrarian; dependent on crop production, livestock keeping, and agriculture labour. The majority of the population is highly vulnerable to shocks, especially the poorest households, and there are a lack of alternative income sources which are further constrained by lack of opportunities and capital.

About 68 % of the population is classified as poor and very poor households (ACF Household Economy Approach Analysis Report 2013 (HEA)) who have limited access to land. A high proportion of very poor households (87 %) are dependent on incomes from casual labour or self-employment; 90 % of these households are reliant on the markets for food purchase all year long. As a consequence of highly insecure cash incomes and a high reliance on food purchased from markets rather than self-produced, very poor households do not typically meet the average daily recommended caloric intake, consuming 1911 kcal/day instead of 2100 kcal/day. Further, a cross-sectional anthropometric nutrition survey carried out by ACF in Dadu in November 2014, showed that the prevalence of global acute malnutrition (GAM) was approximately 14.3 % (95 % CI 10.8–18.7 %) in children 6–59 months. This is expected to be higher during the summer lean season (June to August), where Dadu also frequently experiences localised flooding and droughts, and high temperatures (above 45 °C). Higher levels of GAM for children from poorer households are also expected.

Study participants

Villages are selected from three agricultural areas sharing similar livelihoods and geography. Households eligible for the study are identified as poor and very poor (according to wealth ranking criteria) and with a child or children aged 6–48 months (at baseline).

Recruitment of participants

In order to optimize the interest in the study and ensure meeting sample size requirements, sensitisation about the REFANI-P study is carried out first at village level with village leaders by Field Officers. The Field Officers also conduct a household assessment (household size and asset ownership) in each village to ascertain eligibility of households. After which data enumerators return to the villages and share the details with the village leaders and then visit identified households to share details of the study and get informed consent before collecting baseline data.

Timeline and procedures

REFANI-P starts with formative research using qualitative methods to help develop quantitative data collection

tools. These are then piloted and data enumerators are trained on the questionnaires and data collection techniques, including use of Open Data Kit (ODK) data collection and anthropometry equipment. Anthropometry training is supported by the ACF nutrition team. The timeline is shown in Table 1.

The interventions

Villages are randomly assigned to 4 groups as follows: 1) intervention group receiving a monthly unconditional cash transfer of 1500 Pakistan Rupees (PKR) ('standard' cash), 2) intervention group - receiving a monthly unconditional cash transfer of 3000 PKR ('double' cash), 3) intervention group receiving a monthly fresh food voucher with a monetary value of 1500 PKR to be exchanged for fresh foods at specified traders ('cash voucher'), 4) comparison group receiving no additional intervention beyond the basic ACF care activities provided to all.

The 'standard' amount of cash represents the amount that was defined in the HEA analysis, to cover a 10 % shortfall in calories by very poor households, and is similar to the amount disbursed by the national Benazir Bhutto Income Support Programme. The cash transfers are disbursed on a monthly basis by mobile banks that travel to a central location for each of the participating villages. The vouchers are disbursed by the ACF team to participating households. Disbursement to targeted mothers from all households starts in June.

Standard care

All villages have access to ACF care which provides outpatient treatment for severe acute malnutrition (SAM) for children 6–59 months, micronutrient supplementation for children less than five years and pregnant and lactating women, and behaviour change communication. Key messages focus on the underlying causes of malnutrition, and showcase mother and child nutrition, exclusive breastfeeding, complementary feeding practices, food and water hygiene, and handwashing and sanitation. Additionally these key messages are again delivered to all study participants in group sessions by REFANI-P research mobilisers every month. The key messages are targeted at the mothers/care takers of the eligible children, although other household members are not excluded from access to key messages.

Outcome measures

Measurements are collected on all study households (household questionnaire) and participants (mother and child questionnaire) at month 0, 1, 2, 3, 4, 5, 6 and 12. Measurements are collected during the week following the receipt of each transfer. The primary outcomes are prevalence of wasting (as measured by WHZ < −2 or the presence of bilateral pitting oedema) and mean WHZ score in children.

Secondary outcomes are:

- in children - cumulative incidence of wasting, prevalence of SAM (<−3 WHZ), low mid-upper arm circumference (MUAC) (<125 mm & < 115 mm), stunting (<−2 height-for-age z-score) and morbidity (mainly diarrhoea, difficulty breathing/cough, malaria)
- in mothers and children - mean haemoglobin concentration (Hb g/dL) and prevalence of anaemia (children: Hb <11.0 g/dL; severe anaemia as Hb < 7.0 g/dL; non-pregnant women Hb <12.0 g/dL; pregnant women Hb <13.0 g/dL)
- in mothers - prevalence of low body mass index (<16, <17, <18.5 kg/m^2), low height (<145 cm) and low MUAC (<230 mm & <210 mm).

Anthropometry

Measures include body weight (kg), height (cm), and MUAC (mm). Weights of both mother and children are measured using the *SECA 874* electronic scale (to 0.1 kg accuracy). Height of children (standing and recumbent) and mothers are measured using the *SECA 250* scale (to 0.1 cm accuracy). Age is assessed by both presentation of birth certificate and recall by the mother/care taker using an events calendar. Standard numerical MUAC bracelets are used to record MUAC to the nearest mm. Measurements are taken twice and the average is recorded. All data enumerators are trained according to international recommendations. Standardised weight-for-height and height-for-age *Z* scores and percentiles are calculated as per the World Health Organisation (WHO) guidelines [19].

Table 1 Timeline for REFANI-P

2015			2016/17
Pre-intervention period		Intervention period	Post-intervention period
Jan/May	May/June	June – November	June +
Formative research, training, piloting & village sensitisation	Recruitment & baseline data collection	Monthly quantitative data collection	Endline data collection
		Qualitative data collection	Analysis
		Cost data collection	Reporting

Biochemical measures

Haemoglobin (Hb) g/dl is measured using the *HemoCue 201+* analyser (to 0.1 g/dl accuracy) and adjusted for pregnancy in women [20].

Process evaluation

The process evaluation focuses on the implementation of the intervention which, together with the theory of change, contextual factors and data collected during the implementation, adds additional understanding of the observed study outcomes. Context data is collected through secondary sources and a community questionnaire and includes data on: supply-side availability and accessibility (e.g. health care, food, water - including cost and distance); local disease environment; social/political environment; cultural decision making and empowerment environment, other interventions that may influence the outcome (e.g. NGO/INGO, government programmes); the indirect impact on the traders and market development (including food price fluctuations).

All quantitative measures and time points are summarised in Table 2.

Qualitative study

Empirical qualitative data are collected during the study period, using key informant interviews, focus group discussions, in-depth interviews and individual case narratives (ICN) of study participants. Information is gathered from purposefully selected key informants such as recipients across intervention groups (as well as non-recipients within the villages), research mobilisers, REFANI and ACF staff, those working in different capacities in health, education, public administration, and people working in other organisations that are present for providing assistance to the local population.

A longitudinal qualitative research tracer study (QRTS) is designed to incorporate time and change into the research process. This is carried out though the in-depth interviews and ICNs. The QRTS documents the dynamics of household behaviours and captures critical moments and processes involved in the changes in recipients' lives as a result of the intervention, and other external events that they are exposed to. The QRTS is particularly helpful in capturing 'transitions' experienced by individuals and families following the intervention, and in understanding factors that support or hinder household development once they stop receiving the intervention.

Economic evaluation

Several data sources are used to assess and analyse the total resource use of each intervention modality, namely ACF accounting data, focus group discussions and interviews with ACF and partner organisation staff, community key informant interviews, focus group discussions with beneficiaries, and survey data. Institutional costs are primarily assessed using accounting data, along with staff interviews. Additional financial costs which are not included in the ACF programme accountancy such as any costs from other institutional partners or other important costs which have been allocated to other programme budgets, etc., are identified through key informant interviews and review of programme documentation.

Societal costs are assessed using qualitative and quantitative methods. Focus group discussions with beneficiaries are conducted to better understand the costs borne by beneficiary households and their communities to be able to receive the transfers. In order to triangulate the data collected on direct and indirect programme costs to beneficiaries, questions relevant to the CEA have been included in the household questionnaire conducted at the 6 month mark of the study.

Staff time allocation is assessed to enable an activity-based costing (ABC) methodology, and to allocate and estimate costs per major program activity.

Sample size

To determine the minimum detectable difference in prevalence of wasting for this sample size the following information was used: 1) estimated prevalence of wasting at baseline (16 %); 2) an estimate of the intra-class correlation coefficient (ICC) from a previous survey (0.02); 3) an estimate of the average cluster size (50); 4) for unequal cluster sizes an estimate of the coefficient of variation of cluster sizes (0.65); 5) significance level at 5 % and power of at least 80 %; 6) a likely dropout rate of 10 %; and 7) the estimated number of eligible children per household (2.2).

The sample size is 632 households per intervention group. This sample size is sufficient to measure a detectable difference of 7 % between the intervention groups and the comparison group post intervention. The sample is powered to detect a 0.19 WHZ difference between the intervention and comparison groups (based on a baseline mean of −1.22 (1.10), from previous data).

Sample size calculations were carried out in Stata (SE version 13.1; StataCorp, Special Edition, College Station, Texas 77845 USA) using the [*clustersampsi*] command.

Randomisation

The unit of randomisation is villages stratified by village size (blocks of small, medium and large) to achieve similar numbers of children in each intervention group of the study and ensure an equal distribution of variables at baseline. Due to the likelihood of tensions between villages being created through a public randomisation process, randomisation is done separately by the principal investigator (PI) putting village names into a bowl and drawing out one at a time to add to an 'intervention' bowl.

Table 2 Outcome measures and intermediary factors and time points for all REFANI-P study groups

			Month						
		Baseline	1	2	3	4	5	6	12
Primary and secondary outcomes									
Child variables	Age (months)	x							
	Sex	x							
	Oedema	x	x	x	x	x	x	x	x
Anthropometry									
Child	Weight (Kg)	x	x	x	x	x	x	x	x
	Height/length (cm)	x	x	x	x	x	x	x	x
	MUAC (mm)	x	x	x	x	x	x	x	x
Mother	Weight (Kg)	x	x	x	x	x	x	x	x
	Height (cm)	x							
	MUAC (mm)	x	x	x	x	x	x	x	x
Biochemical (mother & child)	Haemoglobin (g/dl)	x						x	x
Child morbidity (presence, frequency, duration)	Diarrhoea	x	x	x	x	x	x	x	x
	Bloody diarrhoea	x	x	x	x	x	x	x	x
	Cough	x	x	x	x	x	x	x	x
	Difficulty breathing	x	x	x	x	x	x	x	x
	Fever	x	x	x	x	x	x	x	x
	Malaria	x	x	x	x	x	x	x	x
	Measles	x	x	x	x	x	x	x	x
	Health seeking	x	x	x	x	x	x	x	x
	Treatment (by whom)	x	x	x	x	x	x	x	x
	Long term health problems	x						x	x
Mother and child questionnaire									
Mother's health	Age (years)	x							
	Perceived physical health	x	x	x	x	x	x	x	x
	Mental health [25]	x	x	x	x	x	x	x	x
	Pregnancy	x	x	x	x	x	x	x	x
Expenditures on child	Food, non-food, medical	x	x	x	x	x	x	x	x
	Source of money	x	x	x	x	x	x	x	x
Child care	Hours spent per week	x	x	x	x	x	x	x	x
Food security	Individual dietary diversity	x	x	x	x	x	x	x	x
Women's empowerment	Autonomy	x						x	x
	Decision-making	x			x			x	
	Social capital	x						x	x
Household questionnaire									
Demographic data		x							
Household hygiene	Soap (access/availability)	x	x	x	x	x	x	x	x
	Hygiene score (from observation)	x	x	x	x	x	x	x	x
	Water storage	x	x	x	x	x	x	x	x
Wealth & employment	Satisfaction with life	x			x			x	x
	Productive assets (own, bought, sold)	x	x	x	x	x	x	x	x
	Other assets	x						x	x

Table 2 Outcome measures and intermediary factors and time points for all REFANI-P study groups *(Continued)*

	Number in employment	x		x	x	x	x	x	x	x
	Income (including sources)	x		x	x	x	x	x	x	x
	Employment status	x		x	x	x	x	x	x	x
	Hours worked per week	x		x	x	x	x	x	x	x
Expenditures	Food, non-food, medical	x		x	x	x	x	x	x	x
Food security	Household hunger	x		x	x	x	x	x	x	x
	Household dietary diversity	x		x	x	x	x	x	x	x
Coping	Causes of stress	x		x	x	x	x	x	x	x
	Coping mechanisms	x		x	x	x	x	x	x	x
	Migration	x		x	x	x	x	x	x	x
	Loans/debts (incl ability to repay)	x		x	x	x	x	x	x	x
	Schooling	x							x	x
Community questionnaire										
Demographic data		x								
Infrastructure & access		x								
Economic		x								
Health & education		x								
Social capital		x								
Process evaluation										
Implementation	Delivery to individuals					x			x	
	Response of individuals					x			x	

Data collection and management

Three Field Officers are supervising 24 teams, each made up of 1 female and 1 male enumerator. Research Mobilisers are available to help the teams in identifying households and sensitisation about the study.

Quantitative data is collected using ODK software which is a freely available application to design surveys for data collection through smart devices and run on android based platforms. The data are exported into password protected MS Excel on a daily basis and sent to the principal investigator. During the study, children identified with WHZ < −2 (using MOYO charts) [21], MUAC <125 mm and/or oedema are referred to the ACF outpatient treatment programme, whilst remaining in the REFANI-P study. Children with a haemoglobin level < 11.0 g/dl, non-pregnant women <12 g/dl and pregnant women <13 g/dl) are referred for Government of Pakistan nearest health facility for standard treatment.

Qualitative data are being collected by a team of three led by a local lead researcher. Interviews are being recorded then transcribed and translated into English within a week of data collection. The focus group discussions relevant to the cost-effective analysis (CEA) are recorded, transcribed and translated into English. The interviews are also recorded and transcribed and translated if deemed necessary during the course of data collection. Relevant data from the impact study are extracted from the quantitative data set in ODK as described above.

Planned statistical analysis

The study will use both quantitative and qualitative data analytical methods and a cost-effectiveness analysis will be done. The key steps for the quantitative data analysis of outcome measurements will be: 1) cluster level analyses using summary measures between the different time points and 2) individual level analyses adjusted for clustering (longitudinal generalised mixed models). Significance will be assessed at $p < 0.05$ level.

Baseline descriptive analyses will be carried out on all independent variables at cluster and individual levels to assess baseline differences between groups. The data will be examined for outliers, normality and missing data. To test the overall effect of the interventions (on an intention-to-treat basis), changes in outcome variables between the different groups over follow-up measurements (6 months and at 1 year) will be compared. The interventions, measurement time and intervention-time interaction will be included as fixed effects in the models. Children, mothers and villages will be included as random effects in the intercept and in the slope of the different repeated measurements. Potential confounders (e.g. sex, age and baseline nutrition status) will be used if there are differences at baseline. Best fit of the data will be assessed using maximum likelihood ratio tests.

We will examine the data to determine the extent and type of differential attrition, and depending on our findings, we may apply an appropriate adjustment to estimates of intervention effect and/or discuss the possible consequences they may have on the reliability of the estimates. Since there will be more than one child per household in many cases, a secondary analysis using one child per household (selected at random) will be carried out to confirm if results are being driven by a smaller number of larger households. A mediation analysis (using seeming unrelated regression methods [22–24]) will be carried out to measure the extent to which increased mediating variables may mediate the relationship between the different interventions and comparison group and wasting. All quantitative analyses will be carried out in Stata 13.1. Z-scores will be generated using the World Health Organisation (WHO) macro for Stata.

Recorded speech or transcribed text, obtained from qualitative research tools, will be analysed using NVivo 10 for windows software (© QSR International Pty Ltd 2014). Qualitative data will be checked and cleaned prior to analysis. Data obtained from different sample groups, as defined by a range of different criteria (e.g. men and women, interventions and controls, etc.) will be contrasted to explore how the different interventions have been perceived and understood and their effect on different participants. The multidimensional analysis is expected to be cross-sectional at each time point to allow analysis between individuals at the same time period, as well as longitudinally capturing each individual and household's narrative. Analysis will be carried out on 3 levels: thematic, semiotic and a narrative analysis.

The data analysis for the cost-effectiveness study will be based on the institutional, beneficiary and community level quantitative and qualitative data sets, and will be done using Microsoft Excel and TreeAge softwares (TreeAge Pro Healthcare. Williamstown: TreeAge; 2012). Qualitative data will be analysed for themes and contextualising factors. Cost-effectiveness ratios will be calculated as incremental cost-effectiveness ratios (ICER), representing the additional cost per improved outcome achieved in one intervention compared to another intervention or to standard care. Other cost metrics, such as cost per beneficiary, cost-transfer ratio, etc. will also be calculated. A sensitivity analysis will be performed to gain insight into cost drivers, and to determine how sensitive the cost-effectiveness results are to a significant but plausible variation in each of the most important cost parameters.

Collaborating organisations

This study is one of three studies that fall under REFANI. The Pakistan study is implemented by the Emergency Nutrition Network (research partner) and Action Against Hunger (operational partner). The research part of the study is funded by the Department for International Development (DFID). Two of the intervention arms (standard cash and fresh food vouchers) are funded by the European Union (EU). The double cash arm is funded by EU Humanitarian Aid and Civil Protection (ECHO).

Discussion

With an increasing interest and use of cash-based programming in humanitarian settings to prevent acute malnutrition, it is of high priority to determine what works best and in what context. The lack of current evidence makes it difficult for decision-makers to ensure the best strategies are used in terms of impact and sustainability. In terms of establishing what works, or doesn't, requires unpacking the theory of change as well as exploring the processes involved in order to determine where the opportunities and barriers lie.

The present study protocol describes a theory informed cluster randomised controlled trial of three cash-based interventions, randomised among villages with the main objective of maintaining and improving the nutritional status of children less than five years of age throughout the lean period and after 1 year in Dadu District. This study will also provide detailed information on both delivery and use of the intervention. The results of this study will furthermore provide robust evidence to help increase understanding of how and why certain modalities of cash transfers work better than others in a defined humanitarian setting. Findings from the cost-effectiveness analysis will contribute to an understanding of which intervention provides greater value for money in this context, and will provide information on resource use of these intervention strategies by both institutions and beneficiary households in Pakistan. Furthermore, this study will provide important evidence to bridge our gap in understanding the cost-effectiveness of cash interventions in achieving nutrition objectives.

Trial status

Recruitment closed. Trial on-going.

Abbreviations

ACF: Action Against Hunger; ABC: Activity-based costing; cRCT: Cluster randomised controlled trial; CEA: Cost-effective analysis; DFID: Department for International Development; ECHO: EU Humanitarian Aid and Civil Protection; ENN: The Emergency Nutrition Network; EU: European Union; GAM: Global acute malnutrition; Hb: Haemoglobin; HEA: Household Economy Approach; ICC: Intra-class correlation coefficient; ICER: Incremental cost-effectiveness ratios; ICN: Individual case narratives; MUAC: Mid-upper arm circumference; ODK: Open Data Kit; PKR: Pakistan Rupees; QRTS: Qualitative research tracer study; SAM: Severe acute malnutrition; WASH: Water, sanitation and hygiene; WHZ: Weight-for-height Z-score; WHO: World Health Organisation.

Competing interests

The authors declare that they have no competing interests.

Author's contributions
BF is principal investigator for the REFANI-P study, conceptualised the design and drafted the paper. MS coordinates the study implementation at field level. CP and LT conceptualised the cost-effectiveness analysis. SP participated in the conception of the study and led the grant application. All authors revised, reviewed and approved the final paper.

Acknowledgements
We would like to acknowledge the REFANI-P study participants and field teams, The Emergency Nutrition Network (ENN), Action Against Hunger USA/Islamabad/Dadu, the REFANI consortium and all the members of the REFANI steering committee, especially Sheila Isanaka and Erin Lentz.

Author details
[1]Independent consultant for the Emergency Nutrition Network, Oxford, UK. [2]Action Against Hunger Pakistan, Dadu, Pakistan. [3]Action Against Hunger USA, New York, USA.

Received: 17 September 2015 Accepted: 29 September 2015
Published online: 12 October 2015

References

1. Hofmann C. Cash transfer programmes in Afghanistan: a desk review of current policy and pracitice. London, UK: HPG Background Paper, ODI; 2005.
2. Scanteam analysts and advisers. We accept cash. Mapping study on the use of cash transfers in humanitarian recovery and transitional response. Oslo, Norway: Report for the Norwegian Agency for Development Cooperation; 2011.
3. Garcia M, Moore CM. The cash dividend : the rise of cash transfer programs in sub Saharan Africa. Washington DC: World Bank; 2012.
4. Holmes R, Bhuvanendrah D. Social protection and resilient food systems: The role of cash transfers. London, UK: Annual Report, Overseas Development Institute; 2013
5. WHO, UNICEF, WFP, UNHCR. Consultation on the Programmatic Aspects of the Management of Moderate Acute Malnutrition in Children under five years of age. Geneva: World Health Organization; 2010.
6. The World Food Programme (WFP). Revolution: From Food Aid to Food Assistance. Innovations inOvercoming Hunger; 2010. http://www.wfp.org/content/revolution-food-aid-food-assistance-innovationsovercoming-hunger.
7. ACF International Network. Implementing Cash-based Interventions: A Guideline for Aid Workers, ACF Food Security Guideline; 2007. http://www.actionagainsthunger.org.uk/resource-centre/online-library/detail/media/implementing-cash-based-interventions-a-guideline-for-aid-workers/.
8. Harvey P, Bailey S. Cash transfer programming in emergencies. Good Practice Reviews. London, UK: Overseas Development Institute; 2011.
9. Bailey S, Hedlund K. The impact of cash transfers on nutrition in emergency and transitional contexts. A review of the evidence, HPG Commissioned Reports. London: Overseas Development Institute; 2012.
10. Levine S, Chastre C. Nutrition and food security response analysis in emergency contexts. HPG Commissioned Paper. London, UK: Overseas Development Institute; 2011.
11. Hoddinott J, Gilligan D, Hidrobo M, Margolies A, Roy S, Sandstrom S, et al. Enhancing WFP's Capacity and Experience to Design, Implement, Monitor, and Evaluate Vouchers and Cash Transfer Programmes : Study Summary. Washington, DC: International Food Policy Research Institute; 2013.
12. Hidrobo M, Hoddinott J, Peterman A, Margolies A, Moreira V. Cash, Food, or Vouchers? Evidence from a Randomized Experiment in Northern Ecuador, IFPRI Discussion Paper 01234. Washington, DC: International Food Policy Research Institute; 2012.
13. Aker JC. Cash or Coupons? Testing the Impacts of Cash versus Vouchers in the Democratic Republic of Congo. Working Paper 320. Washington, DC: Center for Global Development; 2013.
14. Margolies A, Hoddinott J. Costing alternative transfer modalities, Journal of Development Effectiveness. 2014; doi:10.1080/19439342.2014.984745.
15. Gentilini U. Our Daily Bread: What is the Evidence on Comparing Cash versus Food Transfers? Washington, D.C: The World Bank Group; 2014.
16. Tan-Torres Edejer T, Baltussen R, Adam T, Hutubessy R, Acharya A, Evans DB, et al., editors. Making Choices in Health: WHO Guide to Cost-Effectiveness Analysis. Geneva: World Health Organization; 2003.
17. Musgrove P, Fox-Rushby J. Cost-Effectiveness Analysis for Priority Setting. In: Jamison DT, Breman JG, Measham AR, Alleyne G, Claeson M, Evans DB, Jha P, Mills A, Musgrove P, editors. Disease Control Priorities in Developing Countries. 2nd ed. Washington, D.C.: The World Bank; 2006.
18. Grant A, Treweek S, Dreischulte T, Foy R, Guthrie B. Process evaluations for cluster-randomised trials of complex interventions: a proposed framework for designand reporting. Trials. 2013;14:15.
19. WHO Multicentre Growth Reference Study Group. WHO Child Growth Standards: Length/height-for-age, weight-for-age, weight-for-length, weight-for-height and body mass index-for-age: Methods and development. Geneva: World Health Organization; 2006.
20. Sullivan KM, Mei Z, Grummer-Strawn L, Parvanta I. Haemoglobin adjustments to define anaemia. Trop Med Int Health. 2008;13:1267–71. doi:10.1111/j.1365-3156.2008.02143.
21. Sikorski C, Kerac M, Fikremariam M, Seal A. Preliminary evaluation of the Moyo chart-a novel, low-cost, weight-for-height slide chart for the improved assessment of nutritional status in children. Trans R Soc Trop Med Hyg. 2010;104(11):743–5.
22. Zellner A. An efficient method of estimating seemingly unrelated regressions and tests for aggregation bias. J Am Stat Assoc. 1962;57:348–68.
23. Baron RM, Kenny DA. The moderator–mediator variable distinction in social psychological research: Conceptual, strategic, and statistical considerations. J Pers Soc Psychol. 1986;51:1173.
24. Preacher KJ, Hayes AF. Asymptotic and resampling strategies for assessing and comparing indirect effects in multiple mediator models. Behav Res Methods. 2008;40:879–91.
25. Kessler RC, Andrews G, Colpe LJ, Hiripi E, Mroczek DK, Normand SL, et al. Short screening scales to monitor population prevalences and trends in non-specific psychological distress. Psychol Med. 2002;32:959–6.

Submit your next manuscript to BioMed Central and take full advantage of:

- Convenient online submission
- Thorough peer review
- No space constraints or color figure charges
- Immediate publication on acceptance
- Inclusion in PubMed, CAS, Scopus and Google Scholar
- Research which is freely available for redistribution

Submit your manuscript at www.biomedcentral.com/submit

BioMed Central
