# Supplementary material for: Impact evaluation of different cash-based intervention modalities on child and maternal nutritional status in Sindh Province, Pakistan, at 6 mo and at 1 y: A cluster randomised controlled trial
Source: PLoS Med. 2017 May 23;14(5):e1002305. doi: 10.1371/journal.pmed.1002305 (PMC5441577; doi:10.1371/journal.pmed.1002305)
Supplement: S2 Text — (PDF) [file pmed.1002305.s005.pdf]

National Bioethics Committee (NBC) Pakistan

Ref: No.4-87/14/NBC-170/RDC/ 2304

Date: ~~January 28~~ Feb 12, 2015

Patron
Minister of State, Ministry of National Health Services Regulations and Coordination

Chairperson
Secretary, Ministry of NHSR&C, Government of Pakistan

Vice Chairperson,
Director General, Ministry of NHSR&C, Government of Pakistan

Secretariat
Pakistan Medical Research Council

Members Ex-Officio

President, College of Physicians and Surgeons of Pakistan

President, Pakistan Medical and Dental Council, President

President, Pakistan Association of Family Physicians

Executive Director, Pakistan Medical Research Council, Member/Secretary

WHO Country Representative

President, Supreme Court Bar Association

DGMS (IS)/Surgeon General Pakistan Army

Director General Health, Punjab

Director General Health, Sindh

Director General Health, Khyber Pakhtun Khwa

Director Health Services, FATA

Director General Health, Balochistan

Director General Health, AJK

Director Health Services, Gilgit Baltistan

Registrar, Pakistan Nursing Council

Members

Prof. Dr. Aasim Ahmad *(Chairman REC)*
Prof. Dr. Farhat Moazam (*Chairperson HCEC*)
Prof. Dr Munir Akhtar Saleemi
Prof. Dr Zafar Hayat
Prof. Dr Abdul Razzaq Sabir
Dr Aamir Mustafa Jafarey
Dr Asmatullah
Dr Mahjabeen Khan
Dr Farah Qadir
Dr Farid Khan

Ms Bridget Fenn
Emergency Nutrition Network UK
C/o Mr Shahid Fazal
Head of department Nutrition,
Health and Research (Co-investigator)
H. No 4, Street 38, Sector F-8/1
Islamabad.

Subject: **A cluster randomised controlled trial of the effectiveness and cost-effectiveness of cash transfer programmes on child nutrition status: study protocol for a randomised control trial (NBC-170)**

Dear Ms Bridget Fenn,

I am pleased to inform you that the above mentioned project has been cleared by "Research Ethics Committee of National Bioethics Committee".

Kindly keep the National Bioethics Committee Secretariat updated with the progress of the project and submit the formal final report on completion.

Yours sincerely

(Prof Dr. Aasim Ahmad)
Chairman
NBC-Research Ethics Committee

NBC Secretariat:
