# Supplementary material for: Impact evaluation of different cash-based intervention modalities on child and maternal nutritional status in Sindh Province, Pakistan, at 6 mo and at 1 y: A cluster randomised controlled trial
Source: PLoS Med. 2017 May 23;14(5):e1002305. doi: 10.1371/journal.pmed.1002305 (PMC5441577; doi:10.1371/journal.pmed.1002305)
Supplement: S3 Text — (PDF) [file pmed.1002305.s006.pdf]

REFANI STUDY – QUESTIONNAIRES

HOUSEHOLD QUESTIONNAIRE

(COMPLETE ONE QUESTIONNAIRE PER PARENT (father or mother) OF ***[NAME(S)]*** IN HH)

NOTE: CODE NAMES IN [ ] ARE STATA CODES – IN BOLD THEY ARE THE NAMES OF COMPOSITE VARIABLES

IDENTIFYING INFORMATION (for enumerators)

Round : [____][____]	[MONTH]	Team number:	[_____][_____]
UC NAME: ____		UC number:	[_____][_____]
Village NAME: ____		Village Number:	[_____][_____]
Head of HH name: ____		HH number:	[_____][_____]

1. How many ***years*** has this family lived in this village? [_____________] [HHYLIV]
2. How many people are there in this household? A household is described as all family members who 'usually live and eat here'. Only include current members and not visitors or family members living elsewhere<br>[____][____] [HHSIZE]
3. How many children between the ages 6-48 months are there in this household? [____][____] [CNUM]<br>*This question is for enumerators to check that they collect data on the correct amount of children*
4. HH contact details (number and address):

Interviewed by:
Who is answering the questions? **CODE BOX #1**[____][____] [WHOANS]

Supervisor Name: ___________________________________ Supervisor Signature: ____________________________

DATE_______/_________/__________DD/MM/YYYY

SECTION 1: HOUSEHOLD DEMOGRAPHY

RECORD INFORMATION FOR **EACH** FAMILY MEMBER. FIRST THE FATHER OF THE CHILD FOLLOWED BY PRIMARY CARER AND THEN THE SELECTED CHILD THEN THE OTHER FAMILY MEMBERS IN ORDER OF AGE - **ONLY INCLUDE PEOPLE WHO ARE CURRENT HH MEMBERS – NOT VISITORS OR FAMILY MEMBERS LIVING AWAY PERMANENTLY.ALSO RECORD DETAILS FOR ANYONE WHO DIED IN THE HOUSEHOLD IN THE LAST 3 MONTHS – CHECK THIS WITH THE QUESTIONS ABOVE ON NUMBER OF FAMILY MEMBERS**

QUESTIONS MARKED * ARE BASELINE ONLY

1	A*	B*	C*	D*	E*	F*	G	
	**Family relation to [NAME]**<br><br>**CODE BOX #1**<br><br>[HHPOS]	**Age - *at last birthday* (years)**<br><br>[HHAGE]<br>**[MATAGE]**	**Sex (M/F)**<br><br>[HHSEX]	**Highest schooling level**<br>***00=None***<br>***01= Primary***<br>***02=Secondary***<br>***03= Informal***<br><br>[HHSCH]<br>**[MOTH_ED]**<br>**[FATH_ED]**	**Read &/or write in Sindhi?**<br><br>***00=No***<br>***01= Read only***<br>***02= Write only***<br>***03=Both***<br><br>[SINDHI]	**Do any of the people listed have a permanent health problem that stops performance of normal daily activities?**<br><br>***00=No***<br>***01= yes***<br>[HHDIS]	**Died (in past 3 months)**<br><br>***00= No***<br>***01= Yes***<br><br>[DIED]	**CODE BOX #1**
1.1	[____][____]	[____][____]	[____]	[____][____]	[____][____]	[____][____]	[____][____]	01 = Father
1.2	[____][____]	[____][____]	[____]	[____][____]	[____][____]	[____][____]	[____][____]	02 = Mother
1.3	[____][____]	[____][____]	[____]	[____][____]	[____][____]	[____][____]	[____][____]	03 = INDEX CHILD 6-48 months
1.4	[____][____]	[____][____]	[____]	[____][____]	[____][____]	[____][____]	[____][____]	04= Child < 6 months
1.5	[____][____]	[____][____]	[____]	[____][____]	[____][____]	[____][____]	[____][____]	05 = Child > 5 years
1.6	[____][____]	[____][____]	[____]	[____][____]	[____][____]	[____][____]	[____][____]	06 = Grandfather (father's side)
1.7	[____][____]	[____][____]	[____]	[____][____]	[____][____]	[____][____]	[____][____]	07 = Grandmother (father's side)
1.8	[____][____]	[____][____]	[____]	[____][____]	[____][____]	[____][____]	[____][____]	08 = Other relative adults >18 years
1.9	[____][____]	[____][____]	[____]	[____][____]	[____][____]	[____][____]	[____][____]	09 = Other relative children < 18 years
1.10	[____][____]	[____][____]	[____]	[____][____]	[____][____]	[____][____]	[____][____]	10 = Non-relative

**2***	Who is the full time carer of [NAME]? **CODE BOX #1**	[____][____]	[CAR]
**3***	Religion of Household<br><br>***01= Muslim 02=Hindu 03=Christian 05=Other***	[____][____]	[HHREL]
**4***	Ethnicity of Head of Household<br><br>***01= Sindhi 02=Muhajir 03= Punjabi 04= Pathan 05=Baloch 06=Other***	[____][____]	[HHETH]

SECTION 2: FACILITIES AND INFRASTRUCTURE

**5***	How long does it take to get there? ***MINUTES***	[______] ***minutes***	[HEALTHMIN]
**6***	How far away is it? Kilometers<br><br>**01 = <1km, 02 = 1-5km, 03 = 5+km**	[____][____] Km	[HDIST]

**7***	What is the **main** source of **drinking water** for the members of this HH that they have used in the last thirty days? ***Only one answer***<br><br>**CODE BOX #2**	[____][____]	[WATER]

**CODE BOX #2**			
01=Standpipe	04=Unprotected dug well	08=Bottled	
02=Hand pump	05=Rain water		
03=Protected dug well	06=River/stream		

**8***	How long does it take to collect water from water point (one way)? ***MINUTES***	[______]***minutes***	[TIMWAT]
**9***	How do you treat water before drinking it?<br>**00=Don't treat 01=Boil 02=Chlorine(Phitki) 03= Fabric 04=leave to sediment**	[______]	[TREWAT]
**10***	Do you store water?<br><br>***01=Yes 00=No* If no skip>>14**	[____][____]	[STORWAT]
**11***	How many days do you store water before it is consumed? In ***days***	[____][____]	[STORTIM]
**12***	May I see the containers please?<br><br>**If no skip>>14**		
**13***	*OBSERVE THE WATER STORAGE CONTAINERS/ CHECK AND SEE IF THEY ARE NARROW NECKED OR COVERED*<br><br>***01=All containers narrow necked or covered 02=Some are narrow necked or covered 03=None are narrow necked or covered***	[____][____]	[CONTWAT]
**14***	What is the MAIN kind of toilet facility used by this household? ***Only one answer***<br><br>***CODE BOX #3***<br><br>***THIS VARIABLE IS USED TO CREATE WEALTH INDEX***	Male(s) [____][____]<br><br>Female(s) [____][____]	[MTOILET]<br><br>[FTOILET]

**CODE BOX #3**			
01=Flush toilet/septic tank	***03=Pit latrine (public)***		
02=Pit latrine (household)	***04=Bush/ditch***		

15	Do you have soap in this household?<br>**(*Ask to see the soap)***<br><br>***00=No 01=Yes, soap 02=Yes, sand 03=Yes, soap or sand***	[____][____]	[SOAP]
16	Have you used soap today or yesterday?<br><br>***00=No 01=Yes***	[____][____]	[SOAPY]
17	***OBSERVATION ONLY:*** *Is there a handwashing device e.g. tap, basin, bucket, sink etc in the compound?*<br>***00=No 01=Yes***	[____][____]	[HYGIEN_SC]<br>[HYGIEN_CAT]
18	***OBSERVATION ONLY:*** *Is the inside of the house swept and clean?*<br><br>***00=Not swept 01=Well swept 02=Partially swept***	[____][____]	
19	***OBSERVATION ONLY:*** *Are there any animal or human faeces around the front of the house (within 3 meters of the front door or within the HH compound?*<br><br>***00=No 01=Yes***	[____][____]	

SECTION 3: WEALTH & EMPLOYMENT

20	On the whole, are you with the life that you lead?<br><br>***00 = Not at all satisfied ; 01 = slightly satisfied; 03 = moderately satisfied; 04 = Very satisfied; 05 = Extremely satisfied***	[____][____]	[PPOV]

**21**	**Items**	***a. How many of these items do you currently own***		***b. How many assets you sell in the past month? 00=none***		***c. How many did you buy in the past month? 00=none***	
21.1	Livestock	[____][____]	[ASSET1]	[____][____]	[ASSETSOLD]	[____][____]	[ASSETBUY]
21.2	Milking Animals	[____][____]	[ASSET2]				
21.3	Poultry	[____][____]	[ASSET3]				
21.4	Land owned (acres)	[____][____]	[ASSET4]				
21.5	Land cultivated (acres)	[____][____]	[ASSET5]				
21.6	Tubewell	[____][____]	[ASSET6]				
21.7	Buffalo	[____][____]	[ASSET7]				
21.8	Cow	[____][____]	[ASSET8]				
21.9	Shoats	[____][____]	[ASSET9]				
21.10	Donkey	[____][____]	[ASSET10]				
21.11	Camel	[____][____]	[ASSET11]				
21.12	Donky Carts	[____][____]	[ASSET12]				
21.13	Plough	[____][____]	[ASSET13]				
21.14	Radio	[____][____]	[ASSET14]				
21.15	Iron (electric)	[____][____]	[ASSET15]				
21.16	Fans (electric)	[____][____]	[ASSET16]				
21.17	Sewing machine	[____][____]	[ASSET17]				
21.18	Video or cassette player	[____][____]	[ASSET18]				
21.19	Chair table	[____][____]	[ASSET18]				
21.20	Watches, clock	[____][____]	[ASSET20]				
21.21	TV	[____][____]	[ASSET21]				

21.22	VCR, VCP, VCD	[____][____]	[ASSET22]			
21.23	Refrigerator	[____][____]	[ASSET23]			
21.24	Air cooler	[____][____]	[ASSET24]			
21.25	AC	[____][____]	[ASSET25]			
21.26	Computer laptop	[____][____]	[ASSET26]			
21.27	Bicycle	[____][____]	[ASSET27]			
21.28	Motor cycle	[____][____]	[ASSET28]			
21.29	Car, truck	[____][____]	[ASSET23]			
21.30	Tractor	[____][____]	[ASSET24]			
21.31	Mobile phone	[____][____]	[ASSET25]			
21.32	Cooking stove	[____][____]	[ASSET26]			
21.33	Cooking range	[____][____]	[ASSET27]			
21.34	Stove/burner	[____][____]	[ASSET28]			
21.35	Washing machine	[____][____]	[ASSET28]			

**22**	How many people are in any kind of wage (including in-kind payments) employment in the household? *NUMBER*	[____][____]	[HHEMPLNUM]

	A	B	C
**23**	What is the **primary** source of income for the family **during the past month**?<br><br>***See CODE BOX #4***<br><br>***If answer is 00 >> Q30***<br><br>[HHINCS1]	What is the **secondary** source of income for the family **during the past month**?<br><br>***See CODE BOX #4***<br><br>[HHINCS2]	**Average Income** received **last 30 days** (local currency)<br><br>[HHINCOME]
	[____][____]	[_____________]	[____][____]

CODE BOX #4	
00 = No source at all	07=Cash for Work/training
01 = Agriculture (sharecropper or tenants)	08 = Remittances
02 = Daily wage labour	09 = Pensions
03 = Small Business	10 = Loans
04 = Enterprise	11 = Debt repayments
05 = Charity	12 = Microfinance
06 = BISP	13 = In-kind

Record the CURRENT employment status and hours worked of each working family member who is currently in employment.

24	A	B	C	D
Family Relation ***(Code Box # 1)***<br><br>[HHPOS]	**Job**<br><br>**CODE BOX #5**<br><br>[JOB]<br>**[JOB_MOTH]**<br>**[JOB_FATH]**	**Employment status**<br>***01=Permanent full time***<br>***02= Permanent part time***<br>***03 = Temporary full time***<br>***04 = Temporary part time***<br><br>[EMPLOY]<br>**[EMPLOY_MOTH]**<br>**[EMPLOY_FATH}**	***Hours*** worked per week (paid and un-paid)<br><br>[HOURSW]<br>**[HHWK_MOTH]**<br>**[HHWK_FATH]**	***Hours*** per week spent on child care<br><br>[HOURSCC]<br>**[HHCC_MOTH]**<br>**[HHCC_FATH]**

[____][____]	[____][____]	[____][____]	[____][____][____]	[____][____][____]
[____][____]	[____][____]	[____][____]	[____][____][____]	[____][____][____]
[____][____]	[____][____]	[____][____]	[____][____][____]	[____][____][____]
[____][____]	[____][____]	[____][____]	[____][____][____]	[____][____][____]
[____][____]	[____][____]	[____][____]	[____][____][____]	[____][____][____]

CODE BOX #5		
01 = Working in agriculture	03 = Skilled manual	
02 = Unskilled manual	04 = Non manual	

SECTION 4: EXPENDITURE

25	How much was spent by the household last month on ..(item)?<br><br>*include purchased, home produced and received as gift*	Amount in last 1 month (local currency)	
A	Cereals	[__________]	[HHEXPF1]
B	Vitamin A Rich Vegetables/Tubers	[__________]	[HHEXPF2]
C	Whitetubers/Roots	[__________]	[HHEXPF3]
D	Dark Green Leafy Vegetables	[__________]	[HHEXPF4]
E	Vitamin A Rich Fruits	[__________]	[HHEXPF5]
F	Other Fruits And Vegetables	[__________]	[HHEXPF6]
G	Organ Meat	[__________]	[HHEXPF7]
H	Flesh Meats	[__________]	[HHEXPF8]
I	Eggs	[__________]	[HHEXPF9]
J	Fish	[__________]	[HHEXPF10]
K	Legumes, Nuts And Seeds	[__________]	[HHEXPF11]
L	Milk And Milk Products	[__________]	[HHEXPF12]
m	Oils And Fats	[__________]	[HHEXPF13]
n	Sweets	[__________]	[HHEXPF14]
o	Spices, Condiments	[__________]	[HHEXPF15]
p	Beverages	[__________]	[HHEXPF16]
q	Wild Foods	[__________]	[HHEXPF17]
r	Red Palm Oil	[__________]	[HHEXPF18]
a	Tobacco (cigarettes, tobacco)	[__________]	[HHEXPN1]
b	Paraffin/kerosene	[__________]	[HHEXPN2]
c	Charcoal, firewood	[__________]	[HHEXPN3]
d	Other utilities (electricity, gas, water/sewerage services)	[__________]	[HHEXPN4]
e	Toilet soap	[__________]	[HHEXPN5]
f	Other toiletries (shampoo, toothpaste, hair cream, etc)	[__________]	[HHEXPN6]
g	Washing powder, laundry soap, detergents	[__________]	[HHEXPN7]
h	Other cleaning expenses, equipment (brushes, shoe polish, etc)	[__________]	[HHEXPN8]
i	Matches, candles	[__________]	[HHEXPN9]
j	Batteries, bulbs	[__________]	[HHEXPN10]
k	Bus fares, taxis	[__________]	[HHEXPN11]
l	Other transport expenses (bicycle, car repair, petrol etc.)	[__________]	[HHEXPN12]
m	Hair-cut and other personal services	[__________]	[HHEXPN13]
n	Books, notebooks, newspapers, stationary, etc (not for school)	[__________]	[HHEXPN14]
o	Communications (phone calls -- fixed and mobile, post office expenses, etc.)	[__________]	[HHEXPN15]
p	Medicines/injections	[__________]	[HHEXPM]
q	Entertainment/visit to hotel	[__________]	[HHEXPE]

Now I want to ask you some questions regarding items you bought in the LAST THREE MONTHS

TO BE ASKED AT BASELINE, 6 MONTHS AND 12 MONTHS ONLY

26	**How much was spent by the household last three months on ..(item)?**<br><br>***include purchased, home produced and received as gift***	**Amount in last 3 months (local currency)**	
A	Adult's clothing	[___________]	[HHEXP3M1]
B	Children's clothing	[___________]	[HHEXP3M2]
C	Clothing material and services	[___________]	[HHEXP3M3]
D	Footwear (including repair costs)	[___________]	[HHEXP3M4]
E	Recreation (toys, cinema, photography, records etc.)	[___________]	[HHEXP3M5]
F	Personal articles (umbrella, watch, lighter, belts, etc.)	[___________]	[HHEXP3M6]
G	Children under five health expenditure (doctor's fees, tests, hospitalization charges, etc)	[___________]	[HHEXP3M7]
H	Adult and other children health expenditure (doctor's fees, tests, hospitalization charges, etc)	[___________]	[HHEXP3M8]

Now I want to ask you some questions regarding items you bought in the LAST YEAR

TO BE ASKED AT BASELINE AND 12 MONTHS ONLY

27	**How much was spent by the household last 12 months on ..(item)?**<br>***include purchased, home produced and received as gift***	**Amount in last 12 months (local currency)**	
A	Textiles (bedsheet, towels, mosquito netting, etc.)	[___________]	[HHEXP12M1]
B	Kitchen equipment (cutlery, pots, plates, small equipment)	[___________]	[HHEXP12M2]
C	Lanterns, lamps, torches	[___________]	[HHEXP12M3]
D	Education (tuition/registration fees, uniforms, textbooks, school supplies, transport to school)	[___________]	[HHEXP12M4]

CHILD EXPENDITURE

Now I am going to ask you how much you spent on [NAME] during the last WEEK OR MONTH (study child/ren)

RECORD SEPARETELY FOR EACH STUDY CHILD IF >1 CHILD
Thinking about the money spent on [NAME's] food

**28**	How much money did you spend on **[NAME'S]** food last **WEEK**?	[__________]	[CHEXPF]
**29**	**Have you spent more money on [NAMEs] food this month compared to last MONTH?**<br><br>***00=No change 01=Increase 02=Decrease***	[____][____]	[CHEXPFINC]
**30**	**What was the MAIN source of this money?**<br><br>**CODEBOX #6**	[____][____]	[CHEXPFS]

**CODE BOX #6**	
01 = Cash transfers	06 = Remittances
02 = FFV	07 = Employment
03 = Savings	08 = Loans
04 = Microfinance	09 = Employment
05 = Sale of assets	10 = Other source

Thinking about the money spent on [NAME's] health

**31**	How much money did you spend on **[NAME'S]** health last **WEEK**?	[_________]	[CHEXPMW]
**32**	How much money did you spend on **[NAME'S]** health last **MONTH**?	[_________]	[CHEXPMM]
**33**	Have you spent more money on [NAMEs] health this month compared to last **MONTH**?<br><br>00=No change<br>01=Increase<br>02=Decrease	[____][____]	[CHEXPMINC]
**34**	What was the MAIN source of this money?<br>**CODEBOX #6**	[____][____]	[CHEXPMS]

Thinking about the money spent on other (non-food) items for [NAME]

**35**	How much money did you spend on **[NAME'S]** non-food items last **MONTH**?	[_________]	[CHEXPNM]
**36**	Have you spent more money on [NAMEs] non-food items this month compared to last **MONTH**?<br><br>00=No change<br>01=Increase<br>02=Decrease	[____][____]	[CHEXPNINC]
**37**	What was the MAIN source of this money?<br>**CODEBOX #6**	[____][____]	[CHEXPNS]

SECTION 5: FOOD SECURITY

**38**	Did you or anyone in your household eat anything (meal or snack) ***outside*** of the home yesterday?<br><br>***00= No 01=Yes***	[____][____]	[HCONOUT]
**39**	During mealtime who in the family gets to eat first?<br><br>***01= Father/males; 02=Mother/females; 03=Children;***<br>***04=All adults first; 05=Children first; 06=Eat together***	[____][____]	[HHFDDIST]

40	**Have you or members of your household consumed [FOOD ITEM] during the past week?**<br><br>**Ask full list of items first, then ask details**	**How much of this food did your HH consume during <u>the last week</u> (7 days) including food purchased, produced, grown or received as payment/gift?**	
		How many days in last 7 days	**[HDDS]**
A	CEREALS	[____][____]	[HHCEREAL]
**B**	VITAMIN A RICH VEGETABLES/TUBERS	[____][____]	[HHVITAVEG]
**C**	WHITETUBERS/ROOTS	[____][____]	[HHTUBER]
**D**	DARK GREEN LEAFY VEGETABLES	[____][____]	[HHDARKGN]
**E**	VITAMIN A RICH FRUITS	[____][____]	[HHVITAFRU]
**F**	OTHER FRUITS AND VEGETABLES	[____][____]	[HHOTHFV]
**G**	ORGAN MEAT	[____][____]	[HHIRONMEAT]
**H**	FLESH MEATS	[____][____]	[HHFLESH]
**I**	EGGS	[____][____]	[HHEGGS]
**J**	FISH	[____][____]	[HHFISH]
**K**	LEGUMES, NUTS AND SEEDS	[____][____]	[HHLEGUMES]
**L**	MILK AND MILK PRODUCTS	[____][____]	[HHMILK]
**M**	OILS AND FATS	[____][____]	[HHOIL]
**N**	SWEETS	[____][____]	[HHSWEET]
**O**	SPICES, CONDIMENTS	[____][____]	[HHSPICE]
**P**	BEVERAGES	[____][____]	[HHBEV]
**Q**	WILD FOODS	[____][____]	[HHWILD]
**R**	RED PALM OIL	[____][____]	[HHPALM]
**S**	Rice	[____][____]	[HHRICE]
**T**	Meals eaten out	[____][____]	[HHOUT]
**U**	Other ready-made foods (biscuits/cakes/pasta/ baby foods)	[____][____]	[HHOTH]

**41**	In the past month: Was there ever no food to eat of any kind in your household because of lack of resources to get food?<br><br>***00= No; 01=Rarely; 02=Sometimes; 03=Often***	[____][____]	**[HHFIAS]**
**42**	In the past month: Did you or any household member go to sleep at night hungry because there was not enough food?<br><br>***00= No; 01=Rarely; 02=Sometimes; 03=Often***	[____][____]	
**43**	In the past month: Did you or any household member go a whole day and night without eating anything because there was not enough food?<br><br>***00= No; 01=Rarely; 02=Sometimes; 03=Often***	[____][____]	

<u>SECTION 6: COPING</u>

TO BE ASKED AT BASELINE, 6 MONTHS AND 12 MONTHS ONLY

**44**	Are there any household members who have left for migration in past six months *(i.e. migration for work)* in the past month?<br><br>***00=No 01=Yes***	[____][____]	[HHMIGR]

	***If yes >> Q45* otherwise SKIP>>Q46**		
**45**	How many people left for migration?	[____][____]	[HHMIGRNO]
**46**	During the past ***MONTH*** has there been a situation whereby you thought you could not cope?<br><br>***00= No 01=Yes***<br>***If No skip >> 48 otherwise >>Q47***	[____][____]	[HHCOPE]
**47**	What was the ***MAIN*** thing your family did to cope with this?<br><br>**CODE BOX #8**	[____][____]	[HHCOPES]

**CODE BOX #8**	
01 = Changed dietary habits	06 = Took children out of school
02 = Sold assets	07 = Sent children to work
03 = Migrated for work	*08 = other, pilot*
04 = Took a loan	
05 = Sold land	

**48**	Have you had to do any of the following?<br><br>***00=No 01=Yes***<br><br>**1. Dietary Change**<br>a. Rely on less preferred and less expensive foods<br><br>**2. Increase Short-Term Household Food Availability**<br>a. Borrow food from a friend or relative<br>b. Purchase food on credit<br>c. Gather wild food, hunt, or harvest immature crops<br>d. Consume seed stock held for next season<br><br>**3. Decrease Numbers of People**<br>a. Send children to eat with neighbors<br>b. Send household members to beg<br><br>**4. Rationing Strategies**<br>a. Limit portion size at mealtimes<br>b. Restrict consumption by adults in order for small children to eat<br>c. Feed working members of HH at the expense of non-working members<br>d. Reduce number of meals eaten in a day<br>e. Skip entire days without eating	1a. [____][____]<br><br>2a.[____][____]<br>2b.[____][____]<br>2c.[____][____]<br>2d.[____][____]<br><br>3a.[____][____]<br>3b.[____][____]<br><br>4a.[____][____]<br>4b.[____][____]<br>4c.[____][____]<br>4d.[____][____]<br>4e.[____][____]	[HHCOPE1a]<br><br>[HHCOPE2a]<br>[HHCOPE2b]<br>[HHCOPE2c]<br>[HHCOPE2d]<br><br>[HHCOPE3a]<br>[HHCOPE3b]<br><br>[HHCOPE4a]<br>[HHCOPE4b]<br>[HHCOPE4c]<br>[HHCOPE4d]<br>[HHCOPE4e]

**51**	Have you taken out a loan in the past month?<br><br>***00=No 01=Yes***<br>***If yes >>52* otherwise SKIP>>53**	[____][____]	[HLOAN]

**52**	If yes what was the main reason for this?<br><br>**CODEBOX #9**	[____][____]	[HLOANR]

**CODE BOX #9**	
01 = Repay debts	07 = Water for animals
02 = Festivals	08 = Community obligations
03 = Funeral	09 = Taxes
04 = Illness	10 = Transport
05 = Extra food	11 = To buy phone credit
06 = Livestock drugs/vet bills	*12 = Other, pilot*

**53**	Do you have any debts?<br><br>***00=No 01=Yes***<br>***If yes >>54* otherwise SKIP>>56**	[____][____]	[HDEBT]
**54**	Are you able to pay this debt **<u>this month</u>**?<br><br>***00=No 01=Yes***<br>***If yes >>55* otherwise SKIP>>56**	[____][____]	[HDEBTREP]
**55**	How will you repay this debt? (Main method)<br><br>**CODEBOX #10**	[____][____]	[HHOWREP]

**CODE BOX #10**	
01 = Income from agriculture	07 = Remittances
02 = Income from daily wage labour	08 = Pensions
03 = Income from small business/enterprise	09 = Extra loans
04 = REFANI CTP	10 = Gift
05 = Other charity	11 = Microfinance
06 = BISP	*12 =other pilot*

<u>SECTION 7: SOCIAL CAPITAL</u>

TO BE ASKED AT BASELINE, 6 MONTHS AND 12 MONTHS ONLY

**56**	Are you or any member of the household a member of any organization?<br>***00=No 01=Yes 88 = refused 99=Don't know***<br><br>**If yes >> 57 otherwise SKIP>>58**	[____][____]	[WORG]
**57**	If yes, ***RECORD ALL THAT APPLY***<br><br>**CODEBOX #11**	[____][____]<br>[____][____]<br>[____][____]<br>[____][____]	[WORG1]<br>[WORG2]<br>[WORG3]<br>[WORG4]

**CODE BOX #11**	
01 = Woman's cooperative	03 = Villager's agricultural society
02 = School management committees (SMC)/Students' parent association	04 = Village development committee

**58**	Are you or any member of the household ***currently*** a beneficiary of any ***<u>other</u>*** programme(s) – ***do not include WINS/REFANI here***	[____][____]	[WPGM]

	00=No 01=Yes 88 = refused 99=Don't know		
**59**	Are you or any member of the household ***currently*** a beneficiary of the Benazir Income Support Programme (BISP)?<br><br>***00=No 01=Yes 88 = refused 99=Don't know***	[____][____]	[BISPNOW]
**60**	Have you or any member of the household received a cash transfer or been a beneficiary of BISP in the past year?<br><br>***00=No 01=Yes 88 = refused 99=Don't know***	[____][____]	[BISPEVER]

END

REFANI STUDY – MOTHER & CHILD QUESTIONNAIRE

Some of these questions are of a sensitive nature and must only be asked by a female enumerator

SECTION 1: IDENTIFYING INFORMATION

**1**	Team number:	[____][____]	
**2**	How many children are there in this HH between the ages 6-48 months? [____][____]	Check this matches with HH questionnaire	
**3**	Child order number; 1, 2 or 3<br><br>***(Record data for youngest child first and code 1, 2, 3 depending on the order they are in age – make sure twins are coded the correct way round)***	[____]	[ORDER]
**4**	Child NAME<br><br>**!! Check again you have the right child and then refer to child by that name – make sure twins are coded the correct way round)**		

**5**	Does **[NAME]** have a certificate of birth?<br><br>If yes recode this **(dd/mm/yyyy); if no skip >> Q6**<br><br>**Enter 77 if the day is unknown**<br><br>**DO NOT LEAVE MONTH AND YEAR EMPTY**	------/------/---------	[CDOB]
**6**	What is **[NAME'S]** age **(in months)**	[____][____] Months	[CAGE]
**7**	What sex is **[NAME]**? ***01=Male 02=Female***	[____][____]	[CSEX]

The following questions marked [*] are asked at BASELINE ONLY

SECTION 2: IYCF & DIETARY DIVERSITY

**8***	Was ***[NAME]*** ever breast fed?<br><br>***01=Yes 00=No 98=Don't know***<br><br>***If no skip >> Q13***	[____][____]	[CHBFEV]

**9***	How long after birth did you first put [***NAME***] to the breast?<br><br>***If respondent reports she put the infant to the breast immediately after birth, circle '000' For 'Immediately'.***<br>***If less than 1 hour, circle '1' for hours AND RECORD'00' hours.***<br>***If less than 24 hours, circle '1' and record number of completed hours, from 01 to 23.***<br>***Otherwise, circle '2' and record number of completed days.***	IMMEDIATELY...... 000<br>OR<br>HOURS..............<br>1 [____][____]<br>OR<br>DAYS....................<br>2 [____][____]	[CHBFPUT]
**10***	At what age did **[NAME]** stop receiving breast milk? ***(in months)***<br><br>***Record 00 if child still receiving breast milk***	[____][____] Months	[CHBFSTOP]
**11**	Is ***[NAME]*** currently being breastfed or receiving breastmilk?<br><br>***01=Yes 00=No***<br><br>***If no skip >> Q13***	[____][____]	[CHBFCURR]
**12**	Did [***NAME***] receive breast milk ***yesterday*** during the day or at night?<br><br>***01=Yes 00=No 98=Don't know***	[____][____]	[CHBFY]
**13**	Did [***NAME***] receive any vitamin/mineral drops yesterday during the day or at night?<br><br>**01=Yes 00=No 98=Don't know**	[____][____]	[CHDROPS]
**14**	Has [NAME] taken any multi-vitamin supplements in the past month?<br><br>**00= No 01=Yes 98=Don't know**<br>***Ask to see what they have***	[____][____]	[CHVIT]
**15**	Did [***NAME***] receive any ORS yesterday during the day or at night?<br><br>**01=Yes 00=No 98=Don't know**	[____][____]	[CHORS]
**16**	Did [NAME] eat anything (meal or snack) outside of the home yesterday?<br><br>**00= No 01=Yes**	[____][____]	[CHCONOUT]
**17**	How many meals or snacks (solid, semi-solid, soft, milk feeds) did [NAME] receive yesterday during the day or at night?<br><br>**GIVE NUMBER**<br>**Do not include breast milk**	[____][____]	[CHMFREQ]
**18**	Is [NAME] receiving any supplementary foods such as Plumpynut? (e.g. CSB+,CSB++ etc.)<br><br>**00= No 01=Yes**	[____][____]	[CHSUPP]

**19**	Is [NAME] receiving any Complementary Food Vouchers?<br><br>**00= No 01=Yes**	[____][____]	[CHFV]

[ASK THE FOLLOWING QUESTIONS TO THE MOTHER FIRST AND THEN ASK ABOUT THE CHILD/REN]

**20**	**Food Group**	**Examples**	***00=No; 01=Yes***	
**20.1**	CEREALS	corn/maize, rice, wheat, sorghum, millet or any other grains or foods made from these (e.g. bread, noodles, porridge or other grain products) + *local foods*	[____][____]	[WCEREAL] [CHCEREAL]
**20.2**	VITAMIN A RICH VEGETABLES/TUBERS	carrots, squash, or sweet potatoes that are orange inside + *other locally available vitamin-A rich vegetables (e.g. pumpkin)*	[____][____]	[WVITAVEG] [CHVITAVEG]
**20.3**	WHITETUBERS/ROOTS	white potatoes, or other foods made from roots (e.g. *bay, turnip*)	[____][____]	[WTUBER] [CHTUBER]
**20.4**	DARK GREEN LEAFY VEGETABLES	dark green/leafy vegetables, including wild ones + *locally available vitamin-A rich leaves e.g. amaranth, spinach, fenugreek leaves*	[____][____]	[WDARKGN] [CHDARKGN]
**20.5**	VITAMIN A RICH FRUITS	ripe mangoes, ripe papaya, + *other locally available vitamin A-rich fruits*	[____][____]	[WVITAFRU] [CHVITAFRU]
**20.6**	OTHER FRUITS AND VEGETABLES	other fruits, bananas, orange, pineapple, including wild fruits; tomato, onion, cabbage, including wild vegetables, okra, cauliflower, brinjal, bottle ground, cluster beans, chilli	[____][____]	[WOTHFV] [CHOTHFV]
**20.7**	ORGAN MEAT	liver, kidney, heart or other organ meats or blood-based foods	[____][____]	[WIRONMEAT] [CHIRONMEAT]
**20.8**	FLESH MEATS	beef, lamb, goat, wild game, chicken, duck, guinea hen/fowl, other birds	[____][____]	[WFLESH] [CHFLESH]
**20.9**	EGGS	chicken, duck, guinea hen or any other eggs	[____][____]	[WEGGS] [CHEGGS]
**20.10**	FISH	fresh or dried fish	[____][____]	[WFISH] [CHFISH]
**20.11**	LEGUMES, NUTS AND SEEDS	beans, peas, lentils, ground nuts or other nuts, seeds or foods made from these	[____][____]	[WLEGUMES] [CHLEGUMES]
**20.12**	MILK AND MILK PRODUCTS	milk, cheese, yogurt or other milk products, powdered milk	[____][____]	[WMILK] [CHMILK]
**20.13**	OILS AND FATS	oil, fats, butter or margarine added to food or used for cooking	[____][____]	[WOIL] [CHOIL]
**20.14**	SWEETS	sugar, honey, sweetened soda or juice, sugary foods such as chocolates, candies, biscuits and cakes	[____][____]	[WSWEET] [CHSWEET]
**20.15**	SPICES, CONDIMENTS	Spices(black pepper, salt), condiments, OR *local examples*	[____][____]	[WSPICE] [CHSPICE]

**20.16**	BEVERAGES	Tea, coffee, soda, juice	[____][____]	[WBEV] [CHBEV]
**20.17**	WILD FOODS	Insects, grubs, snails	[____][____]	[WWILD] [CHWILD]
**20.18**	RED PALM OIL	Foods containing red palm oil	[____][____]	[WPALM] [CHPALM]
**20.19**	OTHER	Soft or semi-soft food not listed above	[____][____]	[WOTH] [CHOTH]

SECTION 3: SOCIAL CAPITAL

QUESTIONS TO BE ASKED AT BASELINE, 6 & 12 MONTHS

**21**			
21.1. Groups and networks	1. If you suddenly needed a small amount of money, how many people beyond your immediate household could you turn to? ***NUMBER***<br><br>2. About how many close friends do you have these days? ***These are people you feel at ease with, can talk to about private matters, or call on for help.***	1.[____][____]<br><br>2.[____][____]	[WSCG]<br><br>[WSCG1]
21.2. Trust and solidarity	3. If you were caring for a child and needed to go out for a while, would you ask a neighbour for help? ***00=No 01=Yes 88 = refused***<br><br>4. Generally speaking, would you say that most people can be trusted, or that you can't be too careful in dealing with people?<br><br>***00=No trust 01=Trust 88 = refused***	3.[____][____]<br><br>4.[____][____]	[WSCT]<br><br>[WSCT1]
21.3. Collective action and cooperation	5. Do you help out a local group as a volunteer?<br><br>***00=No 01=Yes 88 = refused***<br><br>6. Have you attended a local community event in the past 6 months (e.g. church fete, school concert, craft exhibition , urs, mela, eid festival **other examples in pilot**)?<br><br>***00=No 01=Yes 88 = refused***	5.[____][____]<br><br>6.[____][____]	[WSCC]<br><br>[WSCC1]
21.4. Social cohesion and inclusion	7. Are there any services where you or members of your household are occasionally denied service or have only limited opportunity to use?<br><br>***00=No 01=Yes 88 = refused***<br><br>8. There are often differences in characteristics between people living in the same village/neighbourhood. For example,	7.[____][____]<br><br>8.[____][____]	[WSCS]<br><br>[WSCS1]

	differences in wealth, income, social status, ethnic or linguistic background/ race/ caste/ tribe. There can also be differences in religious or political beliefs, or there can be differences due to age or sex.<br><br>To what extent do any such differences characterise your village/neighbourhood? ***01= very great extent; 02 = great extent; 03= average; 04 = small extent; 05 = very small extent***		
21.5. Information and communication	9. In the past month, how many times have you made or received a phone call?<br>***NUMBER***<br><br>10. What are your three main sources of information about what the government is doing (*pilot - such as agricultural extension, workfare, family planning, etc.).*<br><br>***CODEBOX #1***	9.[____][____]<br><br>10[____][____]	[WSCI]<br><br>[WSCI1]

**CODE BOX #1**	
01 = Relatives	05 = Work
02 = Neighbours	06 = Local newspaper
03 = Friends	07= FM Radio
04 = NGO	

SECTION 4: AUTONOMY

BASELINE, 6 & 12 MONTHS

**22**	Who in your family usually makes the decision on the following matters?<br>**CODEBOX #2**<br><br>1. Your own health<br><br>2. Large household purchases<br><br>3. Daily household purchases<br><br>4.Visits to family, friends, or relatives<br><br>5. Food to be cooked each day<br><br>6. Number of children and when<br><br>7. Joining organisations<br><br>8. Children's marriage	[____][____]<br><br>[____][____]<br><br>[____][____]<br><br>[____][____]<br><br>[____][____]<br><br>[____][____]<br><br>[____][____]	[WDEC1]<br>[WDEC2]<br>[WDEC3]<br>[WDEC4]<br>[WDEC5]<br>[WDEC6]<br>[WDEC7]

**CODE BOX #2**	
01 = themselves (respondent)	***04= someone else***
02= husband/partner	***05= respondent and someone else jointly***
03= respondent and husband/partner jointly	0***88=refused***

EXTRA QUESTIONS ASKED AT M12

**23**	Who in your family usually made the decision on the following matters? **CODEBOX #2**<br><br>1. How the intervention was utilised<br><br>2. Who collected the intervention<br><br>3. Who went to the market/shops to use it?	[____][____]<br><br>[____][____]<br><br>[____][____]	[WDEC9]<br>[WDEC10]<br>[WDEC11]

SECTION 5: MOTHER'S HEALTH (PHYSICAL AND MENTAL)

**24**	In the past month: Have you been suffering from any illness that has caused you to rest more than usual?<br><br>***00= No 01= Yes, but not too bad 02=Yes, much more***	[____][____]	[WHEALTH]
**25**	In the past month: Have you taken any deworming tablet?<br>***00= No 01= Yes***	[____][____]	[WDEWORM]

**26**	**HOW YOU HAVE BEEN FEELING IN THE LAST FOUR WEEKS**		
00= None of the time, 01= A little of the time, 02= Some of the time, 03= Most of the time, 04= All of the time			
A	In the last four weeks, about how often did you feel tired out for no good reason?	[____][____]	[K10]
B	In the last four weeks, about how often did you feel nervous?	[____][____]	
C	In the last four weeks, about how often did you feel so nervous that nothing could calm you down?	[____][____]	
D	In the last four weeks, about how often did you feel hopeless?	[____][____]	
E	In the last four weeks, about how often did you feel restless or fidgety?	[____][____]	
F	In the last four weeks, about how often did you feel so restless you could not sit still?	[____][____]	
G	In the last four weeks, about how often did you feel depressed?	[____][____]	
H	In the last four weeks, about how often did you feel that everything was an effort?	[____][____]	
I	In the last four weeks, about how often did you feel so sad that nothing could cheer you up?	[____][____]	
J	In the last four weeks, about how often did you feel worthless?	[____][____]	

	**HOW THESE FEELINGS MAY HAVE AFFECTED YOU IN THE LAST FOUR WEEKS.**		
K	In the last four weeks, how many days were you TOTALLY UNABLE to work, study or manage your day to day activities because of these feelings?	[____][____]<br>Number of days	[WFEELINGS1]
L	[Aside from those days], in the last 4 weeks, HOW MANY DAYS were you able to work or study or manage your day to day activities, but had to CUT DOWN on what you did because of these feelings?	[____][____]<br>Number of days	[WFEELINGS2]
M	In the last 4 weeks, how many times have you seen a doctor or any other health professional about these feelings?	[____][____]<br>Number of consultations	[WFEELINGS3]

N	In the last 4 weeks, how often have physical health problems been the main cause of these feelings?	[____][____]	[WFEELINGS4]
00= None of the time, 01= A little of the time, 02= Some of the time, 03= Most of the time, 04= All of the time			

SECTION 6: CHILD'S HEALTH

		27	28
	Has ***[NAME]***had any of the following in the past 2 weeks	***00=No; 01=Yes***<br><br>**If no >> next illness**<br>**If no to all >>Q33**	Has ***[NAME]*** been ill for more than 14 days?<br><br>***00=No, 01=Yes***
A	**Diarrhea**<br>***(More than 3 watery stools a day)***	[____][____]<br>[CHDIA3]	[____][____]<br>[CHDIA3P]
B	**Blood in stools**	[____][____]<br>[CHDIABLD]	[____][____]<br>[CHDIABLDP]
C	**High fever**	[____][____]<br>[CHFEVER]	[____][____]<br>[CHFEVP]
D	**Cough**	[____][____]<br>[CHCOUGH]	[____][____]<br>[CHCOUNP]
E	**Very fast or difficult breathing**	[____][____]<br>[CHCOUGHDF]	[____][____]<br>[CHCOUDFP]
F	**Serious loss of appetite**	[____][____]<br>[CHAPPT]	[____][____]<br>[CHAPPTP]
G	**Vomiting everything**	[____][____]<br>[CHVOM]	[____][____]<br>[CHVOMP]
H	**Convulsions**	[____][____]<br>[CHCONV]	[____][____]<br>[CHCONVP]
I	**Unconsciousness**	[____][____]<br>[CHCONSCI]	
J	**Extreme lethargy**	[____][____]<br>[CHLETHAR]	[____][____]<br>[CHLETHARP]
K	**Measles**	[____][____]<br>[CHMEAS]	
L	**Malaria**	[____][____]<br>[CHMALA]	

**29**	Did you seek medical advice for ***[NAME]*** during the past 2 weeks for any of the illnesses above?<br><br>***01=Yes 00=No***	[____][____]	[CHSEEK]

	if yes > Q30, if no SKIP >>Q33		
**30**	Where did you go for treatment FIRST?<br><br>**CODEBOX #3**<br><br>**If you went to a second place please answer the next question; otherwise skip to Q35**	[____][____]	[CHTREAT1]
**31**	Where did you go for treatment SECOND?<br><br>**CODEBOX #3**	[____][____]	[CHTREAT2]

**CODE BOX #3**		
01=Hospital	06=Pvt. health centre/clinic	11=Drug peddler
02=Public clinic	07=Pvt. Pharmacy	12=Traditional practitioner
03=Integrated Health centre	08=Pvt. Doctor	13=Family member or friend
04=Public mobile clinic	09=Pvt. mobile clinic	
05=Pvt. Hospital	10=Market/shop	

**32**	Did **[NAME]** sleep under a mosquito net last night?<br>**00= No 01=Yes**	[____][____]	[MOSQNET]
**33**	Did **[NAME]** receive a deworming tablet in the last year?<br>***00= No 01=Yes***	[____][____]	[DEWORM]
**34**	Did you attend ANC before you had [NAME] ?<br>***00= No 01=Yes***<br><br>**If yes Q36 if no skip>>**	[____][____]	[CHANC]
**35**	Did you attend ANC before you had [NAME] ?<br>***00= No 01=Yes***	[____][____]	[CHANCVISIT]

**36**	**Does [NAME] have any long term health problems?**<br><br>***01=Yes 00=No***<br><br>**Do not include seasonal diseases**	[____][____]	[CHRONIC]
**37**	**Is NAME currently taking any medications for any of these diseases?**<br><br>***01=Yes 00=No***	[____][____]	[CHRONMED]

SECTION 7 MOTHER/CARER ANTHROPOMETRY/HEAMOGLOBIN

BASELINE, MONTHLY, 12

38	Is the mother pregnant?<br><br>***00=No 01=Yes 99=Don't know***<br><br>***If Yes DO NOT take weight - skip >> Q38***	[____][____]	[WPREG]

BASELINE, MONTHLY, 12

39	Mother's Weight (in Kg)<br><br>**Do not take weight if mother is pregnant**	[____][____] . [____]Kg	[WMWT]
40	Child/ren's Weight(s) (in Kg)	[____][____] . [____] Kg	[CWT]

BASELINE ONLY FOR MOTHER

41	Mother's Height 1 **(in cm)**	[____] [____][____] . [____] cm	[WMHT]
42	Mother's Height 2 **(in cm)** ***check***	[____] [____][____] . [____] cm	

BASELINE, MONTHLY, 12

43	Child Height / length **(cm)**<br><br>***If child <2yrs or if age unknown <87cm measure length***	[____] [____][____] . [____] cm	[CHT]
44	Child Height / length **(cm)** ***check***	[____] [____][____] . [____] cm	

BASELINE, MONTHLY, 12

45	Mother's MUAC (in cm)	[____][____] . [____] cm	[WMUAC]
46	Child MUAC **(in cm)**<br><br>**Refer child to CMAM if MUAC <11.5CM!**	[____][____] . [____] cm	[CHMUAC]

**47**	Child Bilateral Oedema<br><br>***01=Yes 00=No***	1. [____][____]	[OEDEMA]

BASELINE, 6 & 12 MONTHS

**48**	Mother's Haemoglobin concentration (in g/dl)<br><br>***REFER MOTHER TO CLINIC IF ;***<br>***NON-PREGNANT WOMEN HB<12 G/DL***<br>***PREGNANT WOMEN HB<13G/DL 1ST AND 3RD TRIMESTER; <13.5 G/DL IN 2ND TRIMESTER***	[____][____] . [____]<br>g/dl\	[WMHB]

**49**	Child Haemoglobin concentration **(in g/dl)**<br><br>***Refer child to clinic ifHb*<11.0 g/dl**	1. [____][____] . [____]<br>g/dl	[CHB]

REFANI STUDY – PROCESS EVALUATION QUESTIONNAIRE
(COMPLETE ONE QUESTIONNAIRE PER **MOTHER** OF ***[NAME(S)]*** IN HH)

Date : _____/_______/___________DD/MM/YYYY	[DATE]	Team number: [_____]	[TNUM]
HH ID<br>***THIS IS PRELOADED – PLEASE CHECK IT IS THE CORRECT NUMBER FOR EACH HOUSEHOLD***			

THE FOLLOWING QUESTIONS ARE ONLY FOR THE **INTERVENTION ARMS ONLY** – THEY MUST NOT BE ASKED TO THE COMPARISON GROUP

1: DELIVERY TO INDIVIDUALS

**1**	Ask the mother ***JUST TO CHECK*** what she got.<br>***DO NOT GIVE HER THE OPTIONS. RECORD HER ANSWER***<br><br>***02=DC, 03=FFV, 04=SC***	[____][____]	[PE_WHAT]
**2**	***Who received (PHYSICALLY COLLECTED) the [CASH] [VOUCHER]***<br><br>***01 = mother 02=her husband 03=other male HH member 04=other female HH member***<br><br>***If 01 skip to Q 4***	[____][____]	[PE_WHOREC]
**3**	***If it was not the mother who collected the transfer was the transfer handed over to the mother?***<br><br>***00=No 01=Yes all 02 Yes some***	[____][____]	[PE_HANDOVER]

These 2 questions to be asked to ALL arms

**4**	***What messages did you receive? RECORD ALL THAT APPLY***<br><br>***01=Nutrition, 02=WASH, 03= IYCF, 04=Health***	[____][____]<br>[____][____]<br>[____][____]<br>[____][____]	[PE_KEYMSG]
**5**	***Did anybody else also receive the key messages?***<br><br>***00 =no 01=her husband 02=other male HH member 03=other female HH member***	[____][____]	[PE_KEY]

SECTION 2:

CASH AND VOUCHER

6	How do you travel TO the distribution point?<br><br>**01=bicycle 02=donkey cart 03 = local transport 04= motorbike 05= on foot**	[____][____]	[PE_TRAVMODE]
7	How long does it take to get there? This is one way and doesn't include waiting time ***MINUTES***	[______] ***minutes***	[PE_TIMETO]
8	How long did you have to wait to collect your transfer? ***MINUTES***<br><br>***(Waiting time includes the entire time from when they are told to arrive (or when they actually arrive) to when they leave)***	[______] ***minutes***	[PE_TIMEWAIT]
9	How do you travel HOME from the distribution site? See Q6 categories	[______]	
10	How long does it take to get home? ***MINUTES***<br>(This is one way and doesn't include waiting time)	[____][____]	[PE_TIMEFROM]
11	Approximately how much money do you (or another member of your household) spend on transport to collect your cash or voucher transfer? ***Rupees***	[_______]Rupees	[PE_COSTCO LL]
12	Are there any informal fees paid to get the transfer (e.g. % HHs paying any "informal fees" to anybody (person to collect cash/FV, bank staff, ACF staff, food vendors (FFV)))<br><br>***00=No 01=Yes***	[____][____]	[PE_INF_FEE]
13	How satisfied were you with the transfer?<br><br>***Satisfaction (1 to 10; 1 = very poor, 10 = very good)***	[____][____]	[PE_SATIS]
14	*BNF security going home*<br><br>**00=Do not feel safe 01=Feel fairly safe 02=Feel very safe**	*[____][____]*	[PE_SECURE]

SECTION 3:

CASH ONLY

15	What was the main use of the cash? Record the main use **CODE BOX #1**<br><br>***Prompt if necessary***<br><br>***If this was the only use then skip to Q20***	[____][____]	[PE_1CASH_USE]
16	What was the second main use of the CASH? Record the main use using **CODE BOX #1**<br><br>**If all the cash was used on Q6 skip to Q8**	[____][____]	[PE_2CASH_USE]
**CODE BOX #1**			

00 = Not spent yet	05= on food for all the family	10= on non-food items for the index children (excl schooling)	15=loaned to family member
01=Saved all	06= on food for all the children	11= on medical items for all the family	16= loaned to non-family member
02=Saved most	07=on food for index children only	12= on medical items for all the children	
03=Paid off debts	08=on non-food items for all the family (excl schooling)	13= on medical items for the index children	
04=Bought productive assets	09= on non-food items for all the children (excl schooling)	*14=schooling*	

**17**	Was the cash transfer amount sufficient to cover needs<br><br>***00=No 01 = Yes***	[____][____]	[PE_SUFFICIENT]

SECTION 4:

VOUCHERS ONLY

**18**	Were the vouchers used as intended (i.e. redeemed in the shop for food items)?<br><br>***00=No 01=Yes***	[____][____]	[PE_FFVUSE]
**19**	Were any of the vouchers sold?<br><br>***00=No 01=Yes all (skip to Q33) 02=Yes some***	[____][____]	[PE_FFVSOLD]
**20**	Were any of the voucher ***items*** sold?<br><br>***00=No 01=Yes all 02=Yes some***	[____][____]	[PE_FFVITEMSOLD]
**21**	Who were the main beneficiaries of the VOUCHER?<br><br>***01=index children only 02= all children 03=all family 04=adults only 05=male adults only 06=female adults only***	[____][____]	[PE_FFVBNF]
**22**	Was the voucher redeemed correctly i.e. were the correct amount of items received?<br><br>***00=No 01=Yes***	[____][____]	[PE_FFVCORR]
**23**	Were the items received of acceptable quality?<br><br>***00=No 01=Yes***	[____][____]	[PE_FFVQUALITY]

**24**	If given the choice would you have preferred cash to the voucher?<br><br>***00=No 01=Yes***	[____][____]	[PE_FFVPREF]

<u>These questions are only for VOUCHER recipients who sold any of their vouchers</u>

**25**	If you sold the vouchers what did you do with the cash?<br><br>**USE CODEBOX #1 Record all**	[____][____]<br>[____][____]<br>[____][____]<br>[____][____]<br>[____][____]	[PE_FFVSOLDWHAT]

**CODE BOX #1**			
00 = Not spent yet	05= on food for all the family	10= on non-food items for the index children (excl schooling)	15=loaned to family member
01=Saved all	06= on food for all the children	11= on medical items for all the family	16= loaned to non-family member
02=Saved most	07=on food for index children only	12= on medical items for all the children	
03=Paid off debts	08=on non-food items for all the family (excl schooling)	13= on medical items for the index children	
04=Bought productive assets	09= on non-food items for all the children (excl schooling)	*14=schooling*	
